# Real-Time Tracking of Photoinduced Metal–Metal Bond Formation in a d^8^d^8^ Di-Iridium Complex by Vibrational Coherence and Femtosecond Stimulated Raman Spectroscopy

**DOI:** 10.1021/jacs.4c18527

**Published:** 2025-03-06

**Authors:** Miroslav Kloz, Jakub Dostál, Atripan Mukherjee, Martin Pižl, Filip Šebesta, Michael G. Hill, Harry B. Gray, Stanislav Záliš, Antonín Vlček

**Affiliations:** † Extreme Light Infrastructure ERIC, ELI Beamlines Facility, Za Radnicí 835, 252 41 Dolní Břežany, Czech Republic; ‡ Department of Inorganic Chemistry, University of Chemistry and Technology Prague, Technická 5, CZ-166 28 Prague, Czech Republic; § Department of Chemical Physics and Optics, Faculty of Mathematics and Physics, Charles University, Ke Karlovu 3, CZ-121 16 Prague, Czech Republic; ∥ J. Heyrovský Institute of Physical Chemistry, Czech Academy of Sciences, Dolejškova 3, CZ-182 23 Prague, Czech Republic; ⊥ Department of Chemistry, Occidental College, Los Angeles, California 90041, United States; # Beckman Institute, California Institute of Technology, Pasadena, California 91125, United States; ∇ Department of Chemistry, Queen Mary University of London, E1 4NS London, U.K.

## Abstract

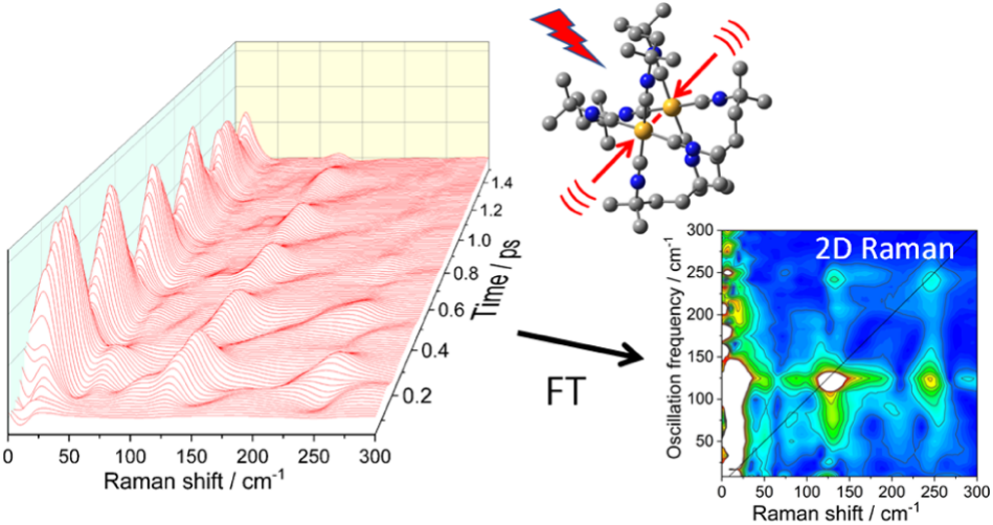

We report real-time dynamics of photoinduced metal–metal bond formation acquired from ultrafast time-resolved stimulated emission and femtosecond stimulated Raman spectra (FSRS) of [Ir_2_(2,5-dimethyl-2,5-diisocyanohexane)_4_]^2+^ (Ir(TMB)) in the region of low-frequency vibrations. Interpretation was supported by impulsive stimulated Raman experiments and time-dependent density-functional theory (TDDFT) calculations. The Ir–Ir stretching frequency doubled on going from ground to the lowest singlet excited state ^1^dσ*pσ, from 53 to 126 cm^–1^, demonstrating Ir–Ir bond formation. Spectral evolution during the first 4 ps after excitation showed extremely large-amplitude coherent oscillations of stimulated emission as well as FSRS signal intensities, which occurred with the excited-state Ir–Ir stretching frequency combined with frequencies of several deformation vibrations and the first Ir–Ir overtone. Corresponding vibrations were observed in FSRS directly but most of them vanished in the first 3 ps, indicating that they belonged to transiently populated hot vibrational states. Fourier transforms of intensity oscillations plotted against FSRS frequencies produced two-dimensional (2D-FSRS) maps with diagonal and off-diagonal features due to Franck–Condon-excited and anharmonically coupled vibrations, some of which acquired Raman intensity through coupling with the Ir–Ir stretch. We concluded that optical excitation impulsively shortens the Ir–Ir distance and increases its stretching force constant, assisted by a simultaneously excited network of coupled deformation modes. The electronically/vibrationally excited system then relaxes through periodic strengthening and weakening of the Ir–Ir interaction and changing conformations of the TMB ligand framework, forming a metal–metal bonded ^1^dσ*pσ state after 4–5 ps.

## Introduction

**Scheme 1:**
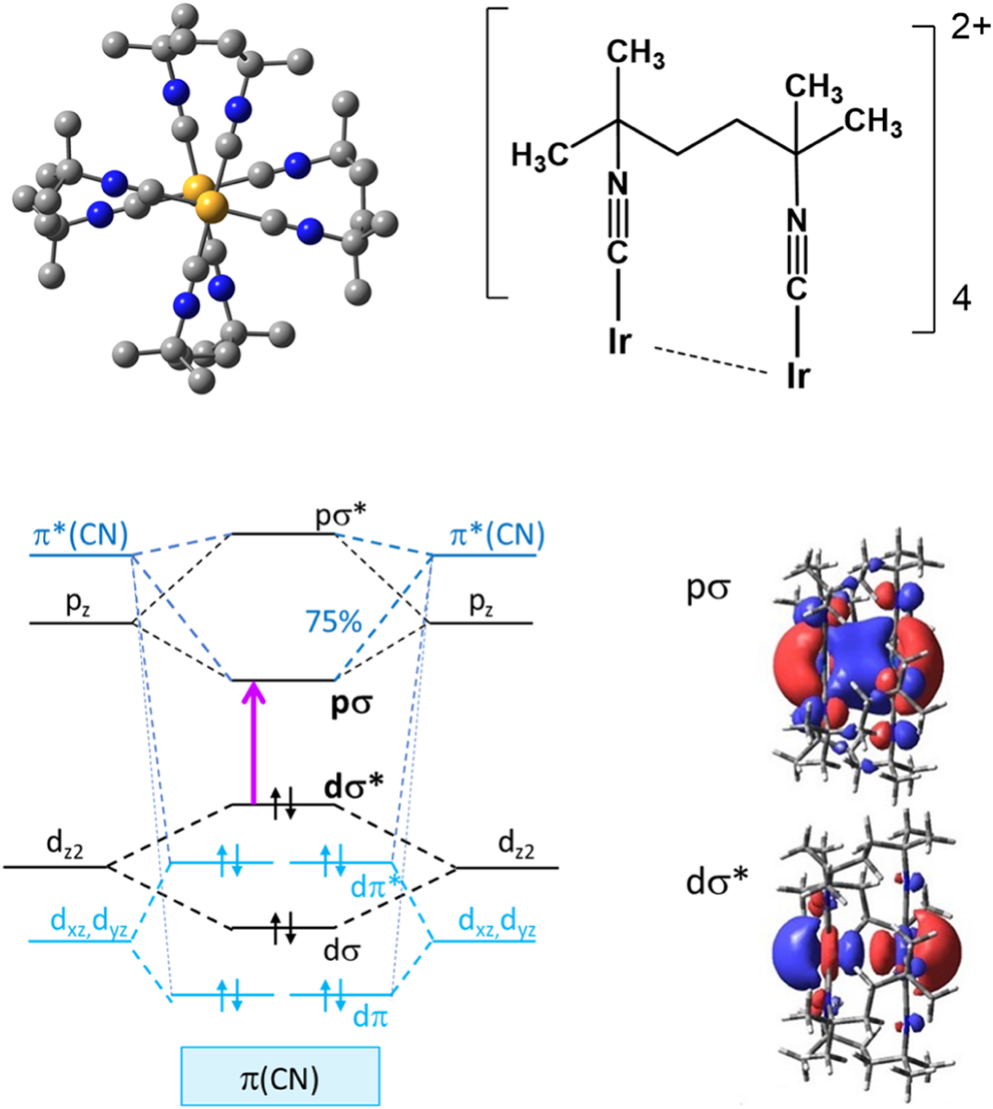
Top: DFT-Calculated Molecular Structure of Ir(TMB) (Ir Orange, C Gray, N Blue). In the Structure, Two Nearly parallel Ir(C≡N−)_4_ Square Planar Units are Rotated with Respect to Each Other by 27.7° (expt^28^ 27°). The Isocyanide Ligands Partially Shield the Ir–Ir Unit from Solvent. Calculated Ground-State Ir–Ir Distance: 3.16 Å (expt^28^ 3.12 Å). There is Strong Dispersion Attraction between the Two Ir(C≡N−)_4_ Planes Coupled with a Very Weak Attractive Ir–Ir Interaction (Bond Order 0.06). Three Higher-Lying Conformers (0.15 < Δ*G*^o^ < 0.24 eV) with Similar Properties were Identified^3^ by DFT (Table S2). Excitation to ^1^dσ*pσ Strengthens Ir–Ir Bonding (Bond Order 0.35) and Shortens the Ir–Ir Distance to 2.87 Å (Twist Angle 27.9°).^3^ Bottom-Left: Qualitative MO Diagram, with Principal σ and π Interactions Indicated in Black and Blue, Respectively. The Violet Arrow Denotes dσ* → pσ Excitation. DFT-Calculated^3^ Ground-State dσ* and pσ Orbitals are Shown on the Right

Outcomes of photochemical reactions are often determined very early after light absorption when excited-state evolution branches between different pathways such as conversion to other electronic states, charge separation, or bond dissociation. Understanding ultrafast excited-state dynamics is thus essential for rational design of light harvesting systems, photocatalysts, or luminophores for display or imaging applications. Binuclear complexes of d^8^ metals (Pt^I^, Rh^I^, Ir^I^) comprise a special class of photoactive systems (d^8^–d^8^) that undergo metal–metal bond formation upon visible-light excitation.^1^ This signature behavior is attributable to electronic excitation from a metal–metal σ-antibonding (dσ*) highest-occupied molecular orbital (HOMO) to a σ-bonding (pσ) lowest unoccupied molecular orbital (LUMO) (Scheme 1).^1−3^ Because of mixing between pσ and ligand π orbitals, the dσ*pσ excited state has partial metal–metal to ligand charge transfer (MMLCT) character that ranges from 53% in [Pt_2_(P_2_O_5_H_2_)_4_]^2–^ (Pt(pop))^2^ to 75% in the binuclear Ir^I^ complex [Ir_2_(2,5-dimethyl-2,5-diisocyanohexane)_4_]^2+^ (Ir(TMB)) discussed herein.^3^

Complexes of the d^8^–d^8^ family exhibit dual photoluminescence and very rich ^3^dσ*pσ photochemistry (reductive and oxidative quenching, radical-like halogen and hydrogen atom abstraction, photocatalytic dehydrogenation, among other reactions).^1,4−6^ Of interest is that relatively long ^1^dσ*pσ lifetimes (ranging from 700 fs to 2 ns depending on structure and solvent) open the way for spin-selective photochemistry. Intramolecular electron transfer to an appended electron acceptor was found to occur from a dσ* → π*(ppy) ^1^MMLCT state of [Pt(ppy)(μ-R_2_pz)]_2_-type Pt(II) dimers (pz = pyrazolyl, ppy = 2-phenylpyridine)^7,8^ and from both singlet and triplet dσ*pσ states of a di-iridium(I) complex [Ir(μ-pyrazolyl)(CO)_2_(PPh_2_Ph-O–CH_2_–acceptor)]_2_.^9^

Irradiating d^8^–d^8^ complexes into dσ* → pσ absorptions with broadband femtosecond pulses generates a superposition of higher metal–metal stretching vibrational states, ν(M-M), creating a coherent vibrational wavepacket that oscillates on the ^1^dσ*pσ potential energy surface. Periodic wavepacket motion along the M–M coordinate (generally, along all Franck–Condon-active modes) is manifested by oscillations of excited-state optical absorption and emission as well as wide-angle X-ray scattering signals. This behavior has been observed for several d^8^–d^8^ systems: Pt(pop) and its perfluoroborated form,^10−12^ a di-iridium complex with a bridging di-isocyano ligand ([Ir_2_(*p*-diisocyanomenthane)_4_]^2+^, Ir(dimen)),^13−15^ [Pt_2_(μ-L)_2_(N^∧^C)_2_] complexes with dσ* → π*(N^∧^C) MMLCT lowest excited states (L = pyrazolyl or 2-hydroxypyridyl derivatives, N^∧^C = 2-phenylpyridine, 7,8-benzoquinoline),^16−21^ as well as unbridged Pt(II) oligomers in solution.^22,23^ Fourier transforms of these oscillations into frequency space revealed a substantial M-M stretching frequency increase upon excitation. For example, ν(Pt–Pt) in Pt(pop) increases from 120 to 149 cm^–1^ in ^1^dσ*pσ,^10−12^ in accord with Pt–Pt distance shortening by 0.24 Å determined^24^ by time-resolved X-ray diffuse scattering. Detailed analyses of vibrational coherence revealed new aspects of d^8^–d^8^ photophysics such as coherence transfer through intersystem crossing (ISC) from singlet to triplet dσ*pσ states^11^ and ratchet-like coherent ISC activation in [Pt_2_(μ-2-hydroxypyridyl)_2_(N^∧^C)_2_] complexes by periodic changes of excited-state energy gaps.^16^

We have investigated the structural evolution and vibrational dynamics associated with ^1^dσ* → pσ excitation in real time by measuring time-resolved femtosecond stimulated Raman spectra (FSRS)^25−27^ of Ir(TMB) (Scheme 1 and Table S1) in three different solvents: acetonitrile (AN), butyronitrile (BN) and tetrahydrofuran (THF). The short Ir–Ir distance (3.12 Å)^28^ and low-energy ^1^dσ* → pσ absorption band^29^ in this d^8^–d^8^ complex indicate strong interaction between the metal atoms. Serendipitously, we observed exceptionally large coherent oscillations and spectral shifts of stimulated emission and Raman signals, indicating that photoinduced Ir–Ir bond formation occurs through large-amplitude structural changes along low-frequency vibrational modes. Combining FSRS with Fourier frequency spectra of signal intensity oscillations produced two-dimensional (2D) Raman maps that revealed the driving role of the Ir–Ir stretching mode (ν(Ir–Ir)) and the coupling pattern of low-frequency vibrations. Together, our dynamic and spectroscopic investigations of electronically excited Ir(TMB) have shed new light on wavepacket motions on a multidimensional anharmonic potential energy surface that results in metal–metal bond formation.

## Results

To investigate the Ir(TMB) response to ^1^dσ* → pσ excitation, we started with femtosecond transient absorption that showed strong coherent oscillations of stimulated emission intensities and pointed to vibrational wavepacket dynamics, setting the stage for Raman experiments. Impulsive Stimulated Raman Spectroscopy identified excited-state Raman features whose dynamics were then studied by Femtosecond Stimulated Raman Spectroscopy that also provided information on vibrational couplings through 2-dimensional spectra. Experimental and computational results are described in detail in the sections below, followed by a discussion section with emphasis on the Ir–Ir bond formation dynamics.

### Time-Resolved Visible Absorption Spectra (TA)

**Figure 1:**
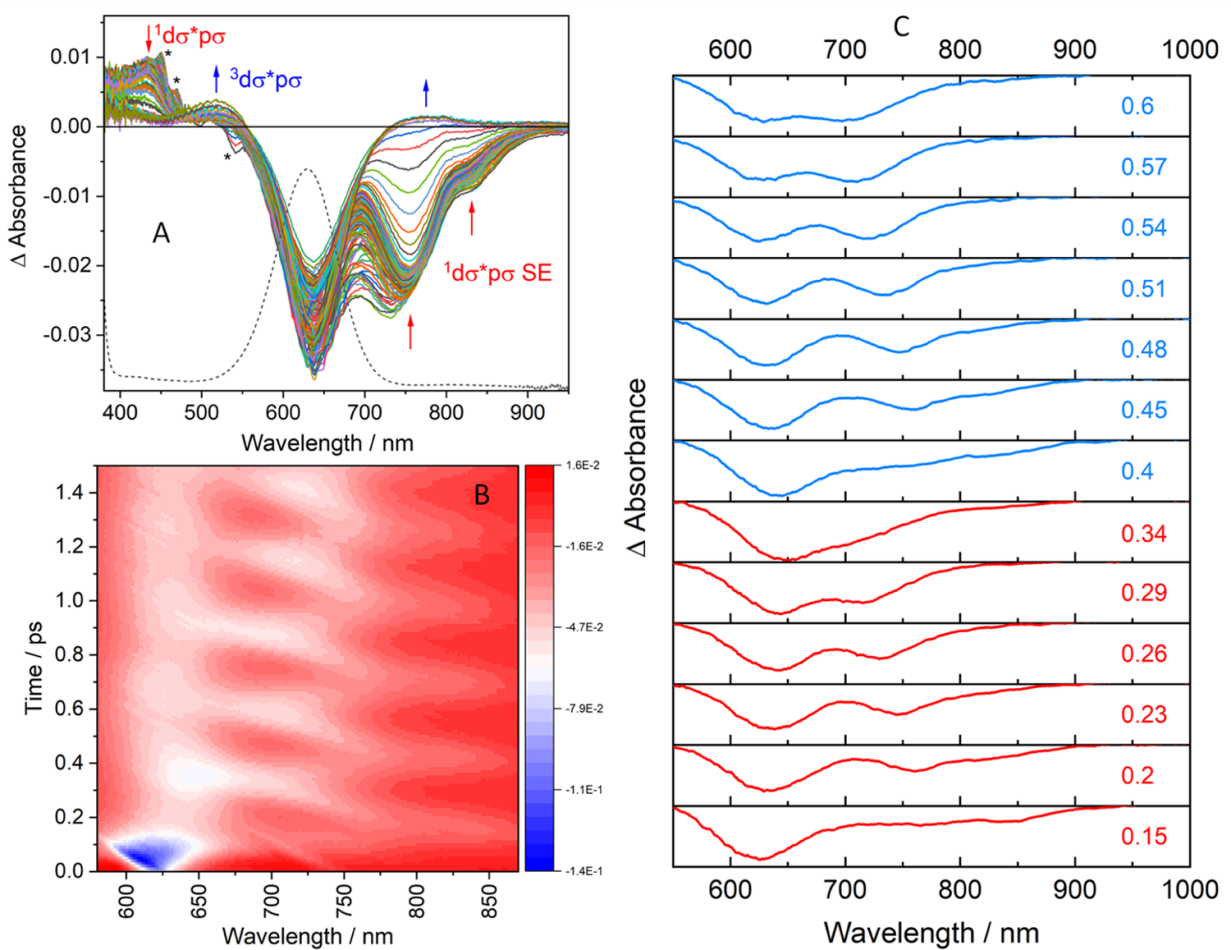
(A): Time-resolved visible absorption (TA) spectra of Ir(TMB) measured over a 0.2–1683 ps time range in butyronitrile (BN). Spectra were recorded every 25 fs between 0.2 and 3 ps and at exponentially increasing time delays after 3 ps. Dashed curve is the ground-state absorption spectrum. (B): Time × wavelength map showing periodic evolution of the GSB/SE spectral pattern over a 0–1.2 ps range. Blue/white colors correspond to strong SE regions. Note the HE-SE dynamic blue shift in each period and overall red shift on going to successive periods, and stretches of stronger SE connecting subsequent SE oscillations between ca. 725 and 760 nm. (C): Selected spectra recorded in BN over the first two oscillations shown in red and blue. Signal intensity (ΔAbsorbance) ranges from −0.068 (bottom) to 0 (top) in each panel. All spectra were chirp-corrected. Time delays are relative, actual *t* = 0 was estimated at +50 fs. Expanded spectra shown in Figure S2C,D. Spectra in acetonitrile (AN) and tetrahydrofuran (THF) are in Figures S4 and S5.

**Figure 2:**
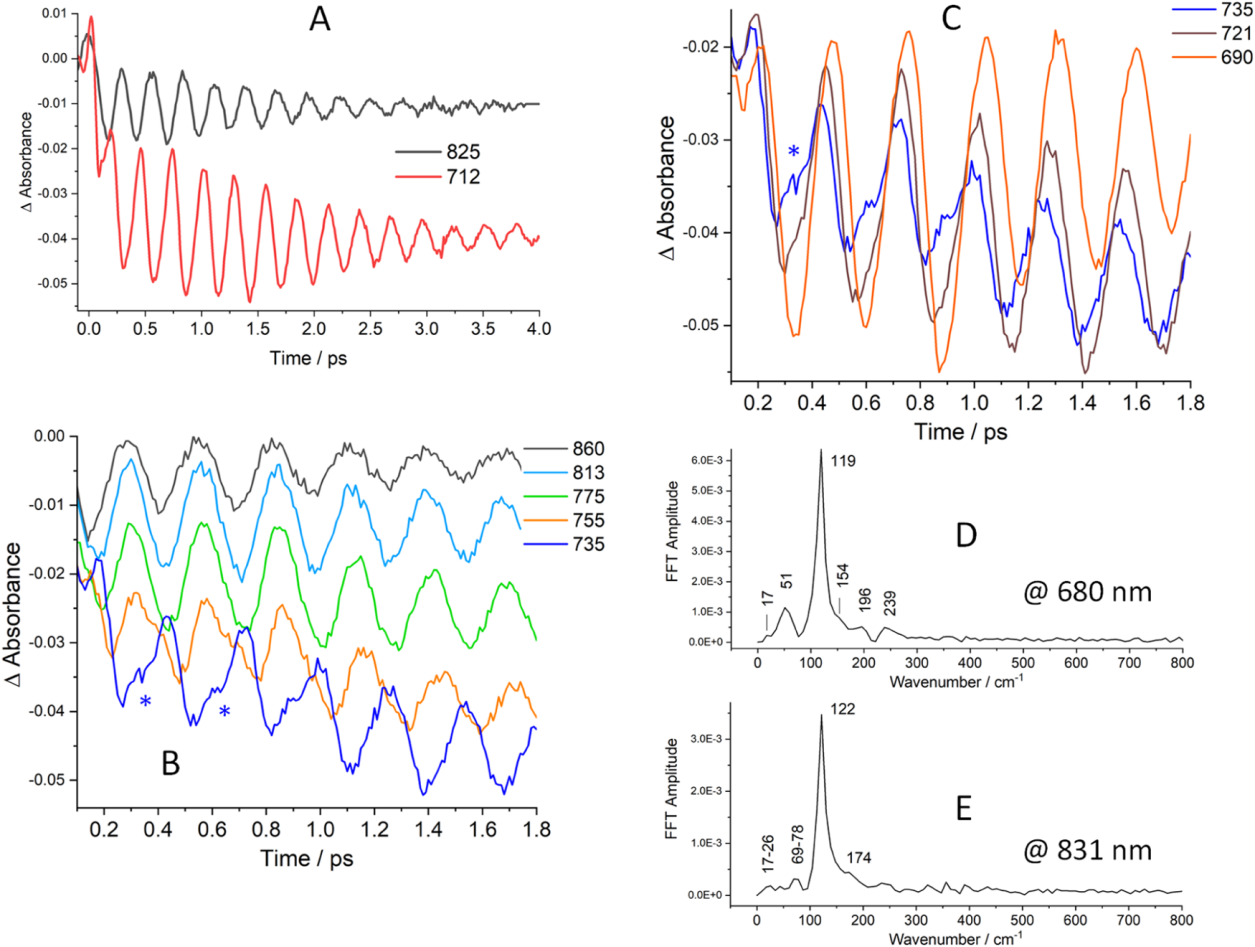
Chirp-corrected time profiles of Ir(TMB) SE intensities in BN measured at various detection wavelengths in 10 fs steps. (A): 4 ps time profiles in the LE-SE (black) and HE-SE (red) regions exhibit opposite oscillation phases. (B): Detail of the LE-SE (860–755 nm) and HE-SE (735 nm) oscillations. A phase-shift occurs between 720 and 755 nm. (C): Detail of the HE-SE region. Oscillations shift to later times with decreasing detection wavelengths. Overtone oscillations are apparent in 735 and 721 nm traces (*). (D, E): Fourier frequency spectra of time profiles at 680 and 831 nm.

Excited-state dynamics of Ir(TMB) were triggered by laser-pulse (∼30 fs fwhm) irradiation into the broad ^1^dσ* → pσ absorption band^3,28,29^ at 629 nm (ε = 11,200 M^–1^cm^–1^, Figures 1A and S1). TA spectra exhibited a ground-state bleach (GSB) at ∼628 nm and broad stimulated emission (SE) that underwent large-amplitude intensity oscillations and periodic spectral shifts spanning the whole range from about 900 nm to the GSB (Figures 1B,C and S2). SE appeared “instantaneously” upon optical excitation as a strongly enhanced GSB signal weakly tailing toward longer wavelengths (Figure 1B, lower-left corner). This high-energy stimulated emission (HE-SE) signal then decayed in intensity while a new low-energy stimulated emission (LE-SE) signal rose in the 760–900 nm range, starting the first fully developed oscillation period. Decay of the LE-SE signal was accompanied by re-emergence of HE-SE as a weak feature at ∼760 nm that grew and shifted to shorter wavelengths toward/into the GSB region while the LE-SE decreased to near-zero intensity. In the second half-period, the HE-SE intensity decreased without shifting to longer wavelengths and LE-SE re-emerged with increasing intensity. This periodic pattern kept repeating itself for the next 4–5 ps with gradually diminishing amplitudes as well as spectral shifts (Figure S3). HE-SE and LE-SE amplitudes oscillated with opposite phases, and a phase shift occurred between 720 and 755 nm (Figures 2 and S6). On going to each successive period, HE-SE slightly narrowed and its maximum moved to longer wavelengths until reaching a final position between 720 and 750 nm at about 3 ps (Figure S3). LE-SE narrowed but retained an average position 810–820 nm. By 5 ps, the HE-SE and LE-SE signals settled into a late-time pattern characterized by a strong band at 736 nm (SE_H_) with a shoulder due to a weaker feature at 813 nm (SE_L_, Figure S2A). In addition to SE, TA spectra showed positive ^1^dσ*pσ excited-state absorption (ESA) at ∼435 nm, with a shoulder at ∼510 nm. Both SE and ESA decayed with a 70 ps lifetime^3^ to ^3^dσ*pσ, characterized by ESA growing at 510 and 720–800 nm that matched previous nanosecond flash-photolysis data.^28^

Signal-intensity oscillations and their Fourier frequency spectra depended on the detection wavelength across the GSB-SE region (Figures 2 and S6 and S7). The GSB blue side (580–600 nm) exhibited weak and short-lived modulation at 53 cm^–1^ (BN), 49–58 cm^–1^ (AN), and much stronger and longer-lived 49–58 cm^–1^ oscillations in THF (Figure S5), matching the ground-state ν(Ir–Ir) vibration at 53 cm^–1^ obtained from spontaneous resonance Raman,^28^ FSRS (Figure S12D), and the DFT-calculated value of 56 cm^–1^. Excited-state SE oscillations were observable from about 600 nm, becoming predominant from ca. 630 nm onward (Figures 2 and S6). The principal contributing frequency shifted from 114–119 cm^–1^ between 630 and 700 nm to 120–124 cm^–1^ at longer wavelengths, matching the main excited-state Raman peak (see below), as well as the TDDFT-calculated excited-state ν(Ir–Ir) frequency of 121 cm^–1^. Fourier spectra indicated further minor contributions from frequencies of about 17–18, 34–35, 51–52, 60–78 cm^–1^, and from several frequencies between 150 and 200 cm^–1^ that appeared as a shoulder on the main 122 cm^–1^ peak (Figures 2 and S7). These additional frequencies are attributable to rotational movements around Ir–Ir and ligand deformations (Tables 1, S3, and Figure S8). In addition, the ν(Ir–Ir) overtone (236–244 cm^–1^), which was a weak feature in Fourier spectra at shorter SE wavelengths (≤660 nm), was stronger between 720 and 765 nm. It (nearly) vanished above 780 nm. The overtone contributions partly filled the minima in time profiles of SE intensities between 720 and 755 nm (Figures 2 and S6), making the oscillations shallow. (This also is manifested by white-pink stretches connecting subsequent SE oscillations in the time × wavelength map (Figures 1B and S3) and saddles between HE-SE ranges in the “waterfall” representation (Figure S2B)). HE-SE oscillations below ca. 740 nm shifted to later times with decreasing detection wavelengths as HE-SE blue-shifted during each period. As a phase flip occurred between 720 and 755 nm (Figure 2B), the red and blue parts of the SE spectrum oscillated with opposite phases. It also is of interest that the SE oscillation amplitude remained constant or slightly increased during the first 1–1.5 ps (depending on the detection wavelength) and only then started decaying.

**Table 1:** Observed and Calculated Vibrational Frequencies of ^1^dσ*pσ Ir(TMB)

Calc’d^a^	SE oscill.^b^	ISRS^c^	FSRS-late^d^	FSRS-early^e^	2D-FSRS^f^	vibration^g^
	(17)		16–18	10–18	13^l^	solute–solvent
29 (B_1_)		(21–26)			25^l^	torsion, peripheral deformation
39 (B_2_)	34–35	(30–34)				torsion, peripheral deformation
52 (A_1_)	51^h^	47^k^, 55^k^	50(−)	∼40–43(−)	50–51^n^	torsion, peripheral deformation
				51–64		
75 (A_1_)	70	68–72	65			torsion, peripheral deformation, bending at N atoms
94 (A_1_)		(85^k^)		68–76(−)	80–75^n^,^o^	torsion, peripheral deformation
				80–105(±)	88^l^, 105^l^	
122 (A_1_)	121^i^	119	126	119–135	128–125^n^	ν(Ir–Ir) stretch
158 (B_2_)	160–170^j^	161		140–165	150–200^l^,^m^	breathing, bending at N, C(C≡N) atoms
185 (B_1_)		174^k^		180		
187 (A_1_)		200				
221 (A_1_)			(210–220)	200–220	208^l^	breathing, bending at N atoms
	236–244	238		237–252(−)	247–241^n^	ν(Ir–Ir) overtone
				240–258		
260 (A_1_)			(265–285)	280	(290)^l^	peripheral deformation, bending at C(C≡N) atoms
281 (A_1_)						
		(357)			(372)^l^	ν(Ir–Ir) second overtone
517 (A_1_)		518			(517)^l^	mixed
551 (B_1_)		(552–561)				

a TDDFT calculated without symmetry constraints for the conformer 4:0 in AN. Symmetry representations were determined by a separate calculation assuming idealized *D*_4_ molecular symmetry. Wavenumbers scaled by 0.956. A detailed list of calculated vibrations is given in Table S3.

b Fourier frequencies of stimulated emission intensity oscillations measured in AN.

c Impulsive stimulated Raman spectra in THF.

d Femtosecond stimulated Raman spectra measured in AN at time delays longer than 5 ps. (−): a negative minimum; (±): alternating maximum/minimum. BN: 45(−), 70–72, 122, 200–300 cm^–1^. THF: weak, estimated at 65–70, 119, 200–300 cm^–1^.

e Ranges over which femtosecond stimulated Raman features evolved during the first ca. 3 ps in AN;

f frequencies identified through 2D-FSRS, see Figure 8 legend.

g Vibrational motions are depicted in Figure S8.

h 51–52 cm^–1^ in BN; 54–56 cm^–1^ in THF.

i 122 cm^–1^ in BN; 120 cm^–1^ in THF. Lower values (99–115 cm^–1^ in THF) were found in the region where SE overlapped with GSB.

j Shoulder on the strong 121 cm^–1^ peak.

k Weakly apparent in the three-pulse experiment.

l From off-diagonal features.

m Encompasses apparent features at 168, 174, 191, 198 cm^–1^.

n Diagonal features.

o Alternative assignment to a cross-peak between two close-lying modes is possible. () denotes very weak signals.

The 435 nm ^1^dσ*pσ ESA signal also showed oscillations dominated by the ν(Ir–Ir) frequency of 121 cm^–1^, with weaker contributions at 33, 55, and 154 cm^–1^.

The nonoscillatory part of HE-SE intensity moved to longer wavelengths with each successive period, which was manifested by the mean HE-SE intensity decreasing with time between 600 and 700 nm and increasing at longer wavelengths over about 3.5 ps with a lifetime of 1.0 ± 0.4 (at 720 nm) and 1.3 ± 0.2 ps (at 760 nm) in BN. LE-SE mean intensity rose with similar kinetics (1.6 ± 0.8 ps at 790 nm) but smaller amplitude. Similar time constants were measured in AN (1.0 ± 0.1 ps rise at 732 nm) and THF (0.9 ± 0.1 ps rise at 741 nm). This dynamic SE red shift is characteristic of vibrational relaxation.

### Potential Energy Surfaces, Vibrational Wavepackets, and Conformational Changes

In the previous section, we demonstrated that Ir(TMB) stimulated emission consists of two components (HE-SE, LE-SE) whose intensities are related by a π phase shift between oscillations. Periodic spectral shifts span at least 4800 cm^–1^ (630–900 nm; 15,900–11,100 cm^–1^) but are not symmetrical as HE-SE appears as a blue-shifting rising band and then uniformly diminishes in the second half-period of each oscillation. SE amplitudes oscillating with opposite phases on the red and blue sides of an SE band are generally attributable to a vibrational wavepacket generated on the excited-state potential energy surface (PES) by an ultrashort (spectrally broad) actinic pulse.^10−12,18,30,31^ In the case of displaced excited- and ground-state PESs, the emission energy and intensity change with changing wavepacket position, owing to energy differences and Franck–Condon overlap between the two states.

**Figure 3:**
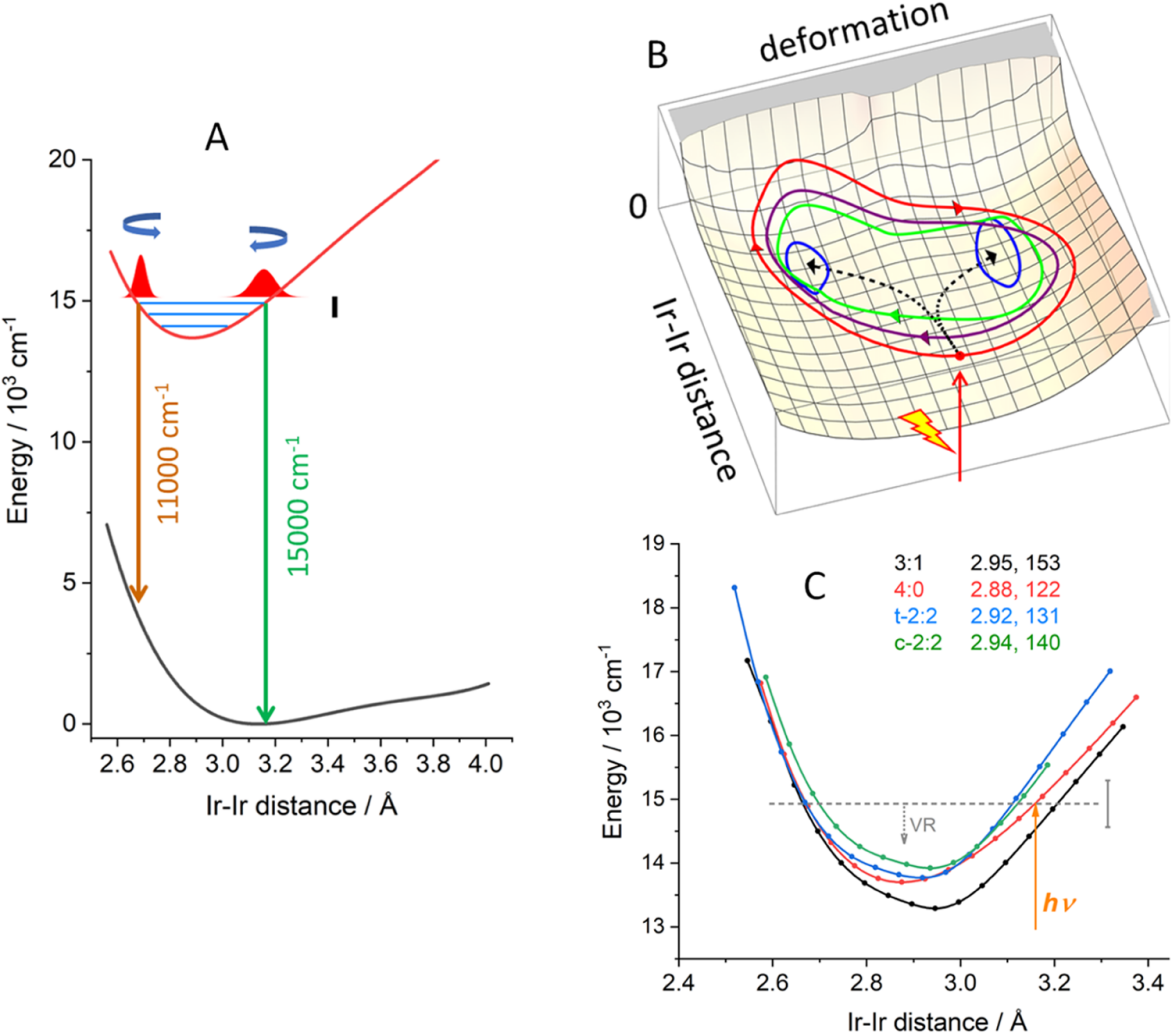
(A): DFT-calculated potential energy curves of the ground (black) and ^1^dσ*pσ (red) states along the Ir–Ir coordinate of the 4:0 conformer (all other structural parameters were optimized at each point). Red Gaussians schematically indicate the vibrational wavepacket at the turning points: right 3.16 Å, 14,930 cm^–1^; left 2.68 Å, 11,060 cm^–1^. Spacing of vibrational levels (blue) is arbitrary: the length of the black bar corresponds to six ν(Ir–Ir) levels that could be excited by a 30 fs actinic pulse and form a wavepacket. (B): Scheme of excited-state evolution on a 2D anharmonic PES. Actinic excitation prepares a wavepacket, which then moves on the PES in descending loops to the two minima (blue). (C): Excited-state PESs of Ir(TMB) conformers. (Structures shown in Table S2). 4:0 (red) is the most abundant GS form excited by the actinic pulse (orange arrow). Gray dashed horizontal line: energy reached by actinic excitation, vertical abscissa: energy range spanned by six ν(Ir–Ir) levels at their equilibrium frequency 122 cm^–1^, downward dotted arrow indicates vibrational relaxation (VR). The inset shows calculated equilibrium Ir–Ir distances (Å) and ν(Ir–Ir) wavenumbers.

The large span of SE spectral shifts in Ir(TMB) can be accounted for by the large relative displacement and different shapes of the ^1^dσ*pσ and ground-state PESs along the Ir–Ir coordinate (Figure 3). Their calculated energy difference varied from 14,930 cm^–1^ at the geometry of the Franck–Condon excitation (3.16 Å) to 11,060 cm^–1^ at the opposite side of the curve (2.676 Å). Wavepacket evolution along the ν(Ir–Ir) coordinate could account for opposite oscillation phases and different band-shapes of LE-SE and HE-SE signals, which approximately correspond to left and right turning points of the wavepacket movement, respectively (Figure 3). Owing to anharmonicity, the wavepacket has different shapes and frequency distributions on the left and right sides of the PES, which could lead to different SE bandshapes (Figure S9). The phase change would be expected^32−35^ to occur when the wavepacket travels over the excited-state PES minimum, which initially was in the 720–755 nm (13,900–13,250 cm^–1^) range, converging to 736 nm (13,580 cm^–1^) at long time delays. However, periodic wavepacket evolution along a single Ir–Ir coordinate cannot fully account for the observed HE-SE spectral evolution in individual oscillation periods. Simulations of a ν(Ir–Ir) wavepacket on a ^1^dσ*pσ PES (Figure S9) showed time-symmetrical behavior whereby the wavepacket shape at every given instant (position) was the same whether it moved from the left to the right (Ir–Ir expansion) or backward (Ir–Ir contraction). In contrast, in the experiment, there was a blue-shifting and growing HE-SE feature in the expanding half-period and only a uniform HE-SE intensity decrease during the contraction phase.

This unexpected behavior suggested that the wavepacket traveled on a two-dimensional (multidimensional) PES over at least two local minima and returned to the starting position along a different path (Figure 3B). In this picture, the wavepacket motion occurs along ν(Ir–Ir) as the principal coordinate, combined with deformation coordinates involving TMB ligands as well as torsional motions around the Ir–Ir bond. (Participation of deformation modes was documented by their frequencies occurring in Fourier spectra of SE oscillations.) The spatial range of the wavepacket motion gradually diminished as vibrational relaxation proceeded simultaneously and the excited population eventually localized in two minima, giving rise to the SE_H_ and SE_L_ bands observed at long time delays.

The presence of spatially and energetically close-lying excited-state local minima was supported by TDDFT conformational analysis that revealed the presence of four conformers with different orientations of the central C–C bond in one or two TMB ligands. (See Table S2 for structures, energies, and notation.) In the GS, larger calculated energy separations predicted that the conformation 4:0 amounted to >97% of the population. In the excited state, their PESs were close to each other, becoming (nearly) degenerate at short Ir–Ir distances. Their equilibrium geometries slightly varied (Figure 3) and their free-energy span (71 meV, 572 cm^–1^) was very small. The occurrence of SE_H_ and SE_L_ bands in late time TA spectra is consistent with the presence of two excited-state conformers.

In summary, stimulated emission experiments pointed to complex Ir(TMB) excited-state dynamics that can be described by a vibrational wavepacket moving on a multidimensional potential energy surface. Fourier analysis of SE intensity oscillations indicated frequencies of vibrations comprising the wavepacket (mostly ν(Ir–Ir) plus several low-frequency deformation modes). In the next sections, we will identify excited-state Raman-active modes by Impulsive Stimulated Raman Spectroscopy by taking advantage of their resonance enhancement by SE and then we will interrogate their dynamics and couplings by time-resolved Femtosecond Stimulated Raman Spectroscopy and its 2-dimensional extension.

### Impulsive Stimulated Raman Spectra (ISRS)^36,37^

**Figure 4:**
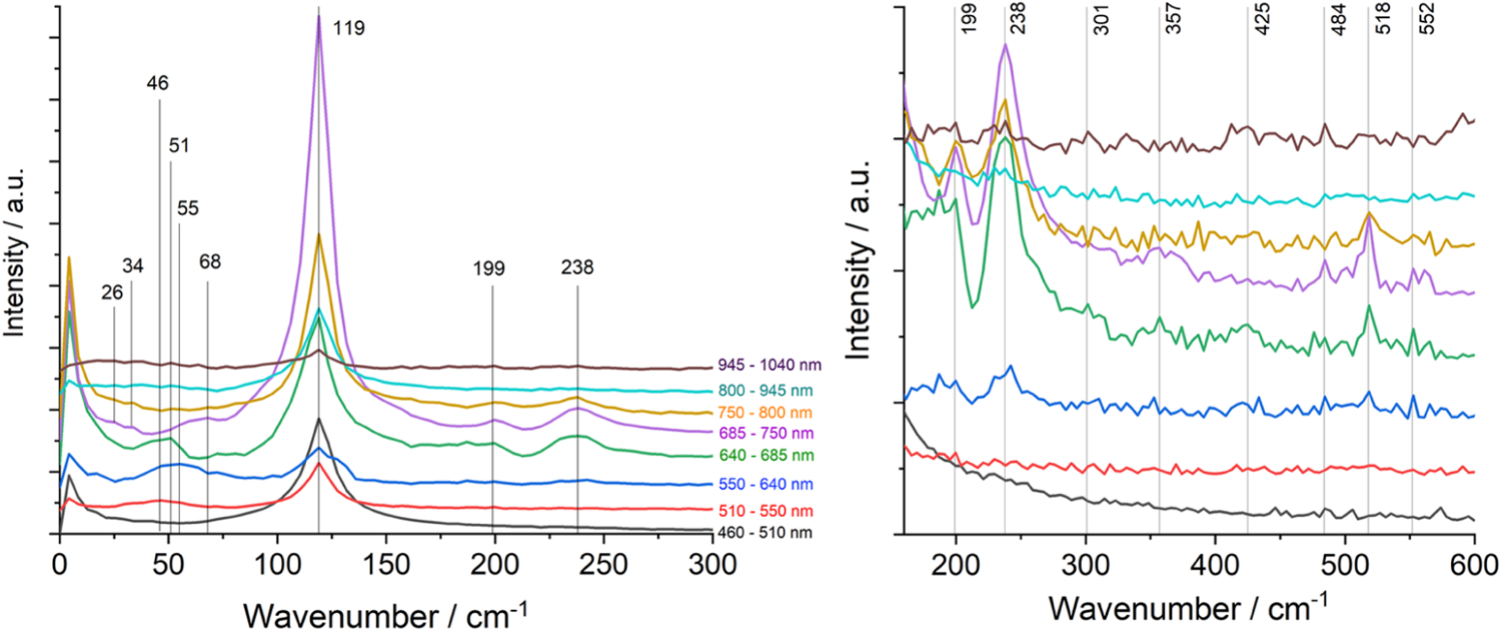
Ir(TMB**)** ISRS in THF solution recorded over different probe-wavelength intervals. Left: ISRS in the 0–300 cm^–1^ range. Right: 150–600 cm^–1^ range with expanded intensity scale. Spectra obtained from the full 460–1040 nm range are displayed in Figure S10.

The sample solution in THF was excited by 4 fs pulses 500–1050 nm broad that acted as a Raman pump and simultaneously converted a fraction of the population to the ^1^dσ*pσ excited state. Impulsively excited Raman modes modulated TA spectra that were probed every 6 fs over the next 8 ps by another 4 fs pulse of much lower energy. ISRS was obtained as a Fourier transform of the oscillatory part of signal time traces measured in selected regions of the TA spectrum. The experiment provided integrated temporal dynamics that resulted in well-defined Raman peaks (Figure 4) whose wavenumbers can be interpreted as averages over the vibrational decoherence time. The peaks that were strongest in detection intervals overlapping with SE (685–750, 750–800, and 640–685 nm) while becoming much weaker at shorter and longer wavelengths were attributed to the ^1^dσ*pσ state: 119 cm^–1^ (ν(Ir–Ir)), its first overtone at 238 cm^–1^, and weaker features at ∼26, 34, 51 (in the 640–685 nm range), 68, 200, 357 (second ν(Ir–Ir) overtone), 518, and 552–561 cm^–1^ (Table 1). Excited-state signals diminished in the 550–640 nm GSB range but increased again between 460 and 510 nm, owing to (pre)resonance with the ^1^dσ*pσ ESA. ISRS determined over the 550–640 nm GSB range exhibited a broad band centered at 55 cm^–1^ attributable to the GS ν(Ir–Ir) mode.^28^ No Ir(TMB) ground- or excited-state ISRS signals were observed above 600 cm^–1^.

ISRS also were measured in a three-pulse configuration, at 300 fs after a 600 nm, 30 fs fwhm actinic prepulse (Figure S10). The spectra were very similar to those obtained without the prepulse, albeit of lower intensity. They exhibited a strong 119 cm^–1^ ν(Ir–Ir) band, together with weak features at 21, 30, 47, 55, 161, and 174 cm^–1^ (Table 1 and Figure S10). (Three-pulse ISRS are complicated by simultaneous oscillations triggered by the 30 fs actinic prepulse and the 4 fs Raman pulse. ISRS intensities will thus depend on their relative timing.)

### Time-Resolved Femtosecond Stimulated Raman Spectra (FSRS)^25−27,38−40^

**Figure 5:**
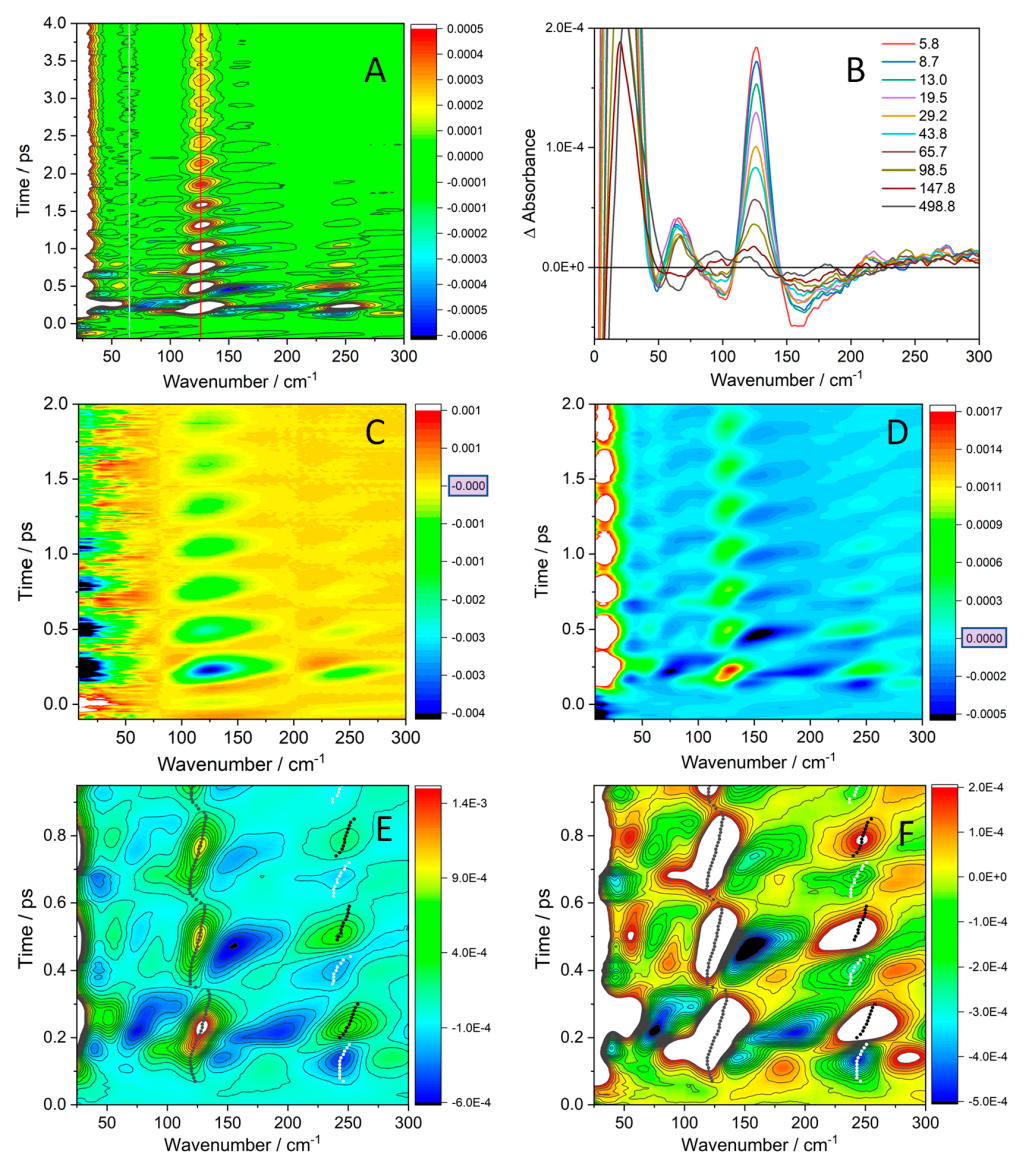
Overview of excited-state FSRS in AN. (A): Time × wavenumber map. White and red vertical lines indicate positions of the late-time bands at 65 and 126 cm^–1^, respectively. (B): Late-time spectra. (C, D): Comparison of Stokes (C) and anti-Stokes (D) FSRS. (E, F): Anti-Stokes FSRS over the first three periods (and beginning of the fourth) shown on different signal-intensity scales. Black (white) points show the positions of apparent maxima (minima) of the ν(Ir–Ir) fundamental and overtone peaks. (Figure S13 shows peak position as a function of time). The deep minimum next to the 126 cm^–1^ ν(Ir–Ir) band evolving between 140 and 200 cm^–1^ results from two overlapping features: a negative signal at ca. 140–165 cm^–1^ (tail of the dispersive-shaped ν(Ir–Ir) feature) and one at ∼180 cm^–1^ that switches between a positive maximum and a negative minimum. (The minimum is clearly seen during the first oscillation).

FSRS were measured together with TA at selected time delays after a 620 nm, ∼ 30 fs fwhm actinic pulse that prepared the Franck–Condon ^1^dσ*pσ excited state and initiated its dynamic evolution. FSRS were obtained using spectrally narrow ∼1.5 ps Raman pump pulse that arrived simultaneously with an ultrashort (∼50 fs) spectrally broad probe pulse. The time delay between the actinic and probe pulses was precisely controlled and varied in 10 fs steps during the first 4 ps after actinic excitation, followed by exponentially increasing steps until 1.68 ns. FSRS signals were superimposed on the transmitted probe pulse spectral profile on blue (anti-Stokes) and red (Stokes) sides of the Raman pump wavelength that was varied between 770 and 795 nm (see SI, Experimental section), which overlapped with Ir(TMB) stimulated emission. Raman features of interest occurred between 0 and 300 cm^–1^ against a strong solvent background that was carefully removed. Figures 5, S11 and S12 provide an overview of FSRS time evolution. Stokes and anti-Stokes spectra displayed the same features with opposite signs, corresponding to Raman gain and loss, respectively.^25,41,42^ (In FSRS, both the Stokes and anti-Stokes regions are dominated by Stokes-like processes. Anti-Stokes FSRS predominantly show features resulting from energy loss of the probe, whereby vibrational energy is transferred to the pump pulse.^25,41,42^ Signals due to hot states could be seen as well.^43−45^ Spectra in the Stokes region arise from energy transfer from the pump to the probe.) Anti-Stokes spectra were better resolved at low frequencies, owing to less scatter and interference from the cell material and solvent. Data and discussion below are based on anti-Stokes FSRS in AN, unless stated otherwise.

**Figure 6:**
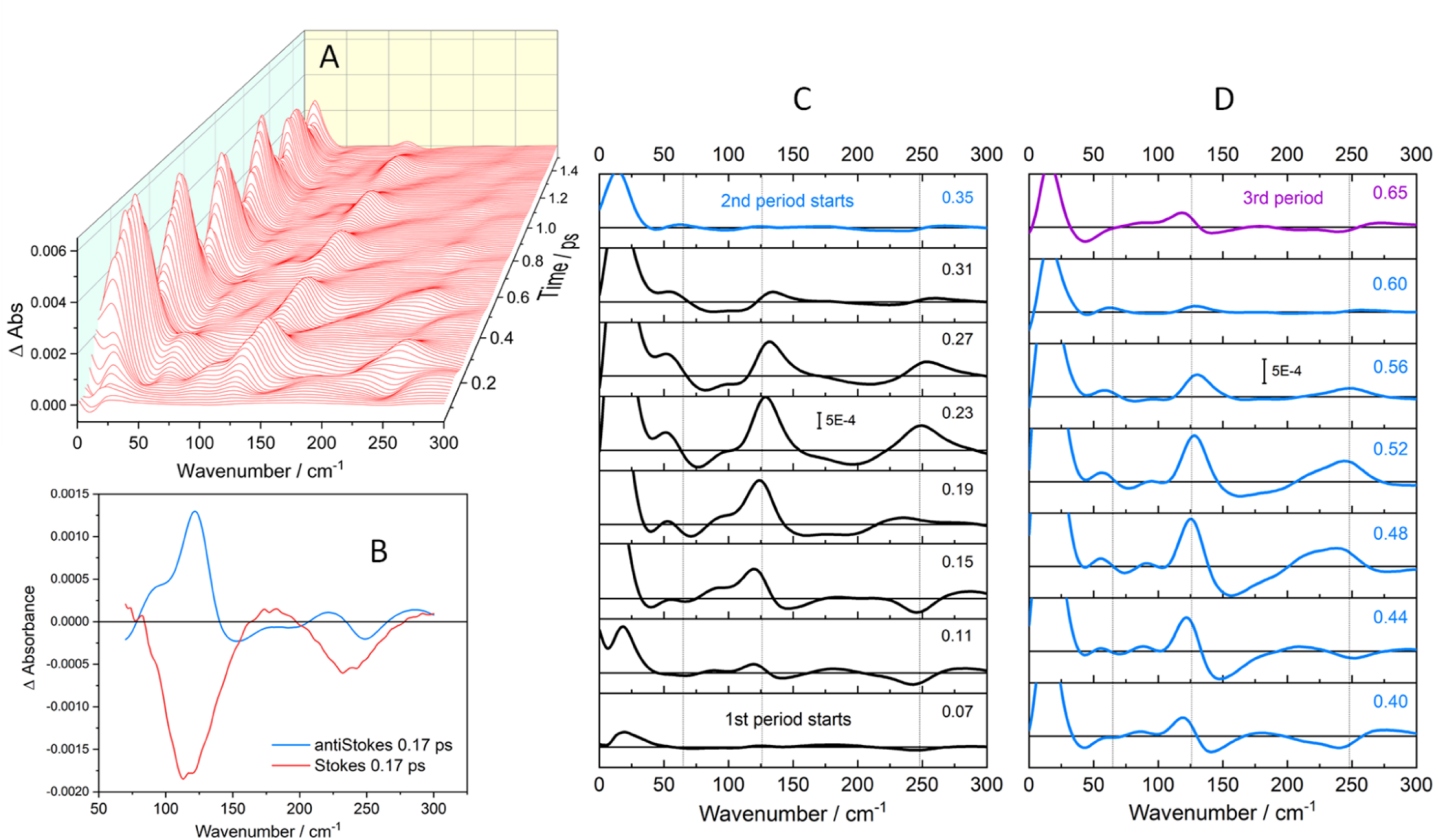
Time-resolved excited-state anti-Stokes FSRS in AN. (A): Spectral evolution from 0.05 to 1.50 ps. (B): Comparison of Stokes and anti-Stokes FSRS measured at 0.17 ps. (C): Selected spectra measured during the first oscillation period (black) and at the very start of the second period (blue, 0.35 ps, top). ΔAbs (*y*-axis) scale: −6.5 × 10^–4^ to 1.7 × 10^–3^. (D): Selected spectra measured during the second oscillation period (blue). The spectrum in violet (0.65 ps, top) belongs to the third period. Δ*A*bs (*y*-axis) scale: −6.5 × 10^–4^ to 1.1 × 10^–3^. Vertical dotted lines indicate peak wavenumbers in late-time spectra: 65 and 126 cm^–1^, and the ν(Ir–Ir) overtone at 248 cm^–1^. See Figures S11 and S12 for more FSRS representations and S19–20 for FSRS in BN and THF.

Excited-state FSRS exhibited large oscillations of peak intensities, positions, and (in some cases) signs in the first 4 ps (Figures 5, 6 and, 7 and S11–S14). FSRS intensities oscillated together with SE, suggesting resonance-enhancement by SE (the ^1^dσ*pσ → ^1^dσ*^2^(GS) transition). Resonance is expected to strongly increase intensities of signals that correspond to totally symmetrical modes.^46^ It complicates FSRS by introducing other photon–molecule interaction pathways that could lead to dispersive band shapes and switching signal signs between negative and positive.^25,45,47−49^

**Figure 7:**
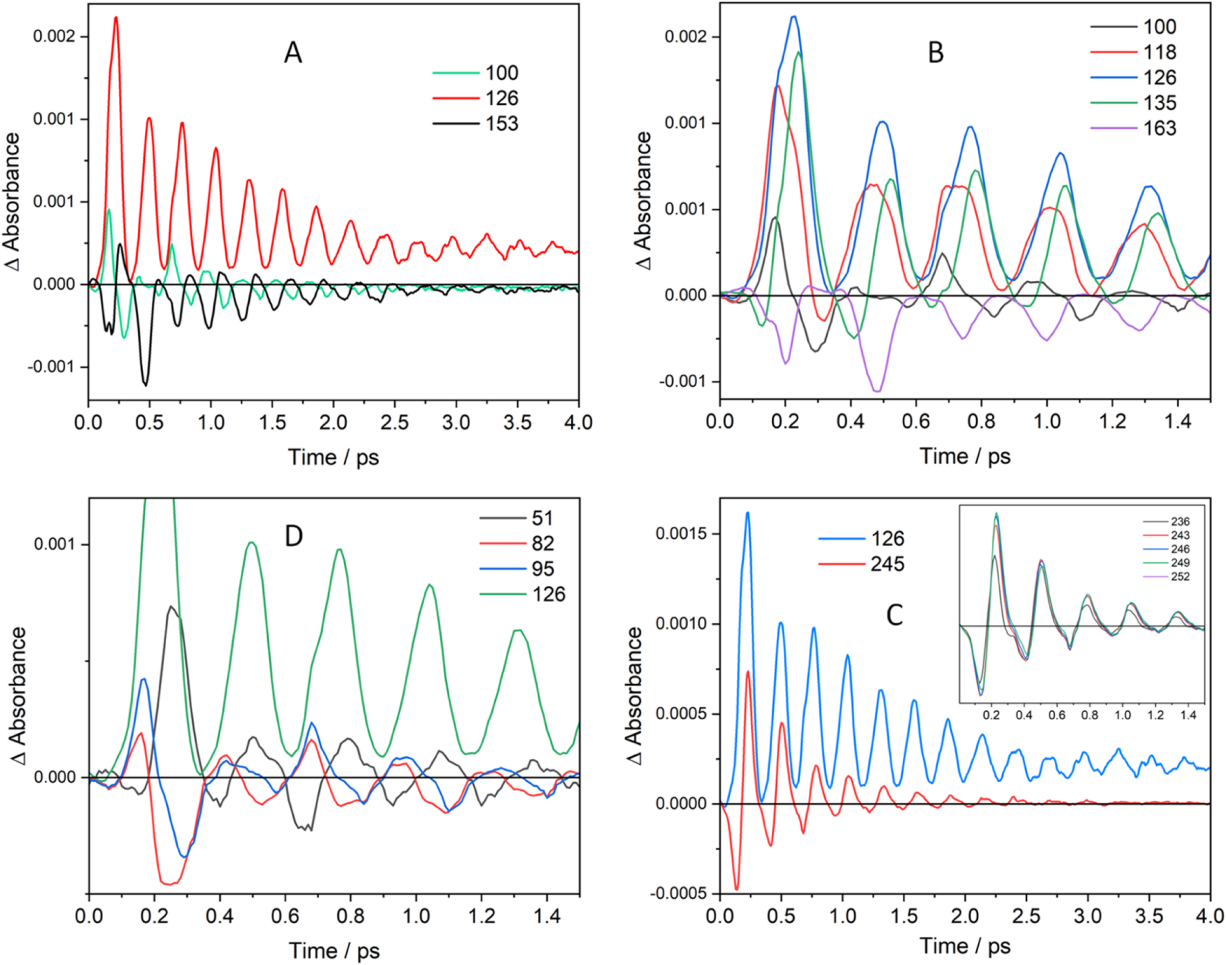
Time profiles of characteristic FSRS signal intensities in AN. (A): Profiles over the first 4 ps. The ν(Ir–Ir) satellites (green, black) vanish by 3.5 ps. (B): Detail of the ν(Ir–Ir) region during the first 1.5 ps. The shift to longer times among 118, 126, and 135 cm^–1^ reflects the dynamic upshift of the ν(Ir–Ir) peak. (C): Comparison of the ν(Ir–Ir) peak (126 cm^–1^) and the overtone at 245 cm^–1^. Inset: selected profiles across the overtone range. (D): Low-frequency features together with the ν(Ir–Ir) peak. Profiles at 82 and 95, and 100 cm^–1^ in B exhibit virtually the same pattern.

“Late-time” FSRS (Figure 5B) were measured at time delays longer than 5 ps, after intensity oscillations abated. The signal decayed commensurately with SE. Spectra included a strong broad positive band at 16–18 cm^–1^ (solute–solvent vibrations), an apparent minimum at ∼50 cm^–1^ (bleached GS ν(Ir–Ir) Raman band), a positive feature at ∼65 cm^–1^ (torsional/(C≡N–C) bending vibration), and a strong excited-state ν(Ir–Ir) band at 126 cm^–1^. The ν(Ir–Ir) band was flanked by weak negative minima at about 100 and 162 cm^–1^, likely due to a background artifact. Very weak positive features were detected at 210–220 and 265–285 cm^–1^. Spectra measured in BN and THF were very similar to those in AN (Table 1—footnote d, Figures S19 and S20). No signals were observed in the range of ν(C≡N) vibrations. The late-time Stokes spectrum in AN exhibited only a weak/broad feature around 120 cm^–1^.

Early time FSRS showed positive and negative features in the 0–300 cm^–1^ range that underwent large-amplitude intensity oscillations with a period of about 270 fs (time profiles in Figures 7 and S14; Fourier spectra in Figures S15–S17), as well as periodically shifting peak positions (Figures 5E,F and S13). Some of these features eventually evolved into long-lived bands seen in the late spectra (16–18, 65, 126 cm^–1^), whereas others decayed and vanished by 3–4 ps.

The ν(Ir–Ir) feature exhibited a dispersive band shape with a positive peak at ∼126 cm^–1^ flanked on its high-wavenumber side by a negative minimum at 140–165 cm^–1^ that vanished by 3–4 ps. The peak wavenumber together with the adjacent minimum showed distinct periodic shifts (Figures 5 and S13). After an initial weak intensity oscillation (0–130 fs, better seen in Stokes, Figures 5C and S11A), the ν(Ir–Ir) peak appeared at the beginning of the first fully developed intensity oscillation at ∼123 cm^–1^ (0.07 ps, anti-Stokes), shifted to 119 cm^–1^ by 0.12 ps, then slowly moved to higher wavenumbers as the peak intensity passed through its maximum, reached 135 cm^–1^ and then abruptly shifted to lower wavenumbers through the region of minimal intensity back to 119 cm^–1^. At this point, the peak started gaining intensity again in the second oscillation (Figure S13). These periodic but not sinusoidal peak-wavenumber shifts continued through the following periods, their range gradually narrowing and eventually converging to 126 cm^–1^ (Figures 5 and S13). The corresponding ν(Ir–Ir) Stokes feature was broad and asymmetric but did not exhibit a dispersive shape. A 140–160 cm^–1^ shoulder was apparent on the high-wavenumber side. The Stokes ν(Ir–Ir) peak dynamically shifted over a broader range (ca. 100–146 cm^–1^) than in anti-Stokes, also undergoing abrupt downshifts in regions of intensity oscillation minima.

The anti-Stokes ∼126 cm^–1^ intensity oscillated with a large positive amplitude that dropped to near zero in the minima (Figures 7 and S14). Oscillations shifted to longer times on increasing the probing wavenumber (118, 126, and 135 cm^–1^), owing to a ν(Ir–Ir) upshift during each period. Fourier spectra revealed a predominant oscillatory frequency of 125 cm^–1^, accompanied by smaller-amplitude contributions at 25, 75, 166–208, and 242 cm^–1^. The negative intensity at 140–165 cm^–1^ oscillated opposite to the ∼126 cm^–1^ peak, so that increasing peak intensity was accompanied by deepening of the adjoining minimum (Figures 7B and S14B). Fourier spectra lacked amplitude in the 140–165 cm^–1^ range while showing a predominant 125 cm^–1^ frequency and weaker contributions at 33–50, 92, 208, and 246–250 cm^–1^ (Figure S16). Both the mean intensity and the oscillation amplitude at 126 cm^–1^ decreased exponentially with a 1 ps time constant after an initial ∼0.3 ps interval. Similar values were estimated in BN: 0.7 ps (mean intensity decay) and 1 ps (osc. amplitude).

The band at 16–18 cm^–1^ was the strongest spectral feature observed over the examined temporal range. It emerged between 50 and 70 fs at ∼20 cm^–1^, quickly moved to 10–14 cm^–1^ in the first period, and then reappeared in the 14–18 cm^–1^ range. Its intensity oscillated with a huge amplitude, in-phase with the ν(Ir–Ir) band (Figure S14A), and eventually evolved into a strong 16–18 cm^–1^ band in late-time spectra. A similar feature was observed in BN at 6–10 cm^–1^. In THF, it was buried in the broad solvent background.

The 65 cm^–1^ band in late-time spectra (excited-state torsion/deformation) emerged from a region initially encompassing several features due to hot torsional/dihedral/deformation modes, namely a 40–43 cm^–1^ minimum and a 51–56 cm^–1^ maximum that then repeatedly switched into a 62–64 cm^–1^ maximum and back. These features periodically shifted positions and changed shapes and signs until about 3.5 ps, when they converged to the 65 cm^–1^ maximum (Figure 5A). Signal intensities between 40 and 51 cm^–1^ oscillated together and were slightly shifted in phase relative to 126 cm^–1^ (Figures 7D and S14C,D; Fourier spectra in Figure S17). The mean intensity at 65 cm^–1^ increased with a time constant of approximately 0.8 ps, much like the vibrational relaxation lifetime determined at 126 cm^–1^.

The ν(Ir–Ir) overtone occurred as alternating minima and maxima that shifted between 240 and 258 cm^–1^ and oscillated in phase with the 126 cm^–1^ peak (Figures 5E,F, S13 and 7C). Intensity oscillations involved the principal frequency of 125 cm^–1^, plus minor contributions at 33, 50, 208, and 241 cm^–1^ (Figure S15). Its mean intensity vanished by ∼3 ps, suggesting that the overtone transition is observable only while a hot molecule is vibrating in the PES anharmonic region.

Transient bands assigned to the vibrational hot molecule also were observed on the low-wavenumber side (80–105 cm^–1^) of the ν(Ir–Ir) band. Initially, the signal appeared as a positive shoulder on the ν(Ir–Ir) band, first increasing with time and then decreasing midway of the ν(Ir–Ir) intensity rise, turning negative (Figures 6C,D, 7B,D and S14D). Signal intensity fluctuated close to zero during the second oscillation period and re-emerged at the start of the third, when it followed the same pattern as in the first period. This seemingly irregular behavior originated from the presence of several medium-amplitude oscillatory frequencies that accompanied the principal 125 cm^–1^ frequency (Figure S17). FSRS features in the 80–105 cm^–1^ range can be tentatively attributed to dihedral and torsional deformation modes (Tables 1 and S3). The signal around 75 cm^–1^ (a torsional/deformation mode) oscillated similarly but its intensity was negative most of the time. Overall, oscillations at 70–105 cm^–1^ had a nearly opposite phase and different Fourier spectra to those at 40–64 cm^–1^, suggesting that they are associated with different vibrations.

### Periodic Shifts of the ν(Ir–Ir) Peak and Vibrational Wavepackets

To obtain information on structural changes induced by excitation, we have used the ν(Ir–Ir) peak wavenumber as a proxy for the frequency of the Ir–Ir stretching vibration despite the complicated FSRS band shape and the investigated dynamics likely being faster than the Raman decoherence time.^25,41,45,49−51^ This is supported by similar peak wavenumbers in late-, early time, Stokes, and anti-Stokes FSRS, and their match with the principal wavenumber in Fourier spectra of SE intensity oscillations.

As outlined above, the ν(Ir–Ir) FSRS peak exhibited periodic but nonsinusoidal dynamics shifts. This behavior accords with the wavepacket model presented in the Potential Energy Surfaces, Vibrational Wavepackets and Conformational Changes section and Figure 3: Increasing ν(Ir–Ir) frequency signaled wavepacket movement toward shorter Ir–Ir distances and a steeper PES. The FSRS peak intensity initially increased as well, reaching a maximum about midway of the Ir–Ir contraction. Reverse wavepacket movement to longer Ir–Ir started as a smooth/slow ν(Ir–Ir) downshift, followed by a sudden drop. This behavior agrees with the looplike wavepacket movement. The excited-state conformation appears to have changed in the region of short Ir–Ir (where the conformers are quasi-degenerate), then the Ir–Ir distance slightly lengthened until the wavepacket quickly returned to the Franck–Condon area of long Ir–Ir and shallow potential within a few tens of femtoseconds. Simultaneous vibrational cooling restricted the spatial range of wavepacket movements and, hence, reduced the spectral shifts. Descent toward the PES minimum also made the system less anharmonic, diminishing Raman intensities of transiently populated hot deformation vibrations, as well as of the anharmonically allowed ν(Ir–Ir) overtone transition.

### 2-Dimensional FSRS

**Figure 8:**
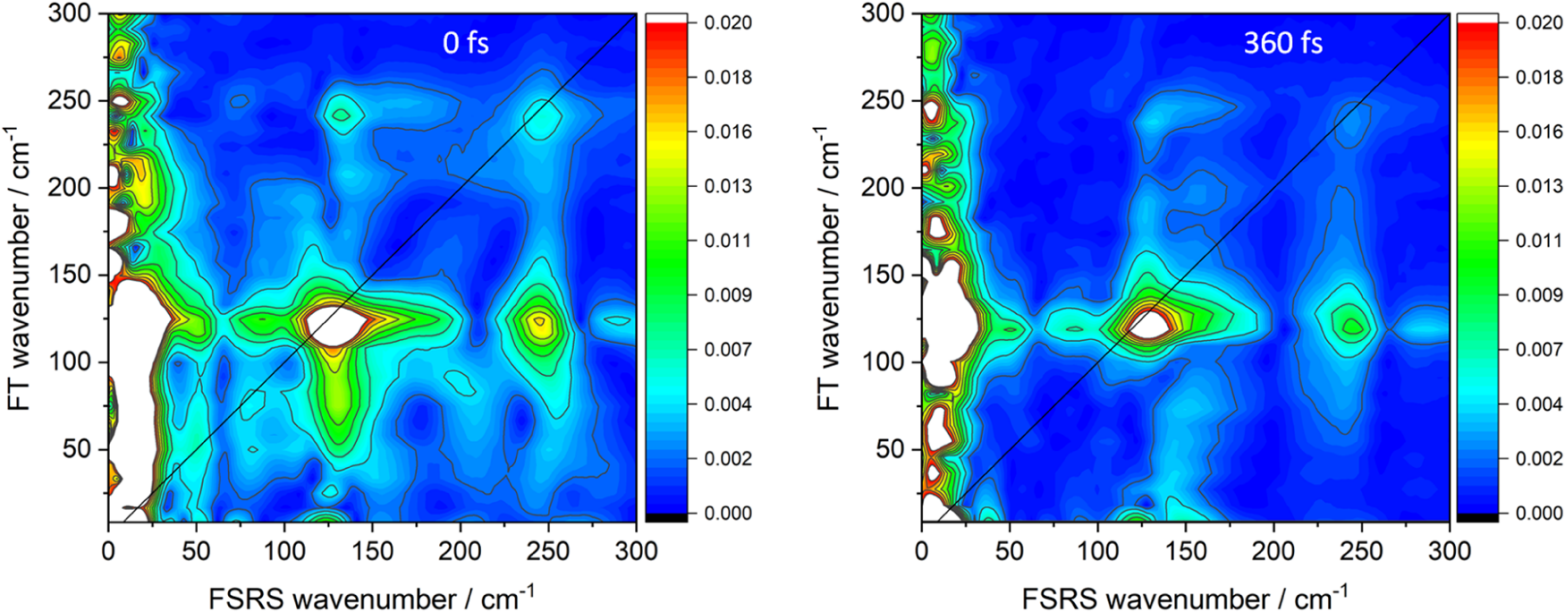
2D-FSRS of Ir(TMB) in AN. The *y*-axis shows wavenumbers of Fourier-transformed 4 ps time-traces of FSRS intensities measured in 1 cm^–1^ steps along the *x*-axis. FT magnitudes are color-coded. FT wavenumber resolution is 8.32 cm^–1^. The diagonal is marked by a black line. The two panels show spectra obtained by starting FT at 0 (left) and 360 fs (right) after the actinic pulse. (Figure S18 shows 2D spectra on different scales and a 60 fs spectrum). Diagonal peaks: 128 × 124 (ν(Ir–Ir), strongest), 247 × 241 cm^–1^ (overtone), 50 × 51, 80 × 75 cm^–1^. Horizontal cross-peaks at the ν(Ir–Ir) FT wavenumber: 13 × 124 cm^–1^ (strongest), 245 × 124 cm^–1^ (overtone), left tail includes 51 × 118 and 88 × 119 cm^–1^ peaks, right tail extending to 200 cm^–1^, and 290 × 124 cm^–1^. Vertical cross-peaks at/around the ν(Ir–Ir) position: 132 × 242 cm^–1^ (strongest, overtone), lower tail encompassing 107 × 51, 130 × (75–80), and 126 × 25 cm^–1^ peaks. The upper tail is branched in the 0 fs map: 114 × 191, 135 × 174, 136 × 208 cm^–1^ changing to a single vertical feature in the 60 fs map with a 122 × 194 cross-peak. The 360 fs map shows cross-peaks at 127 × 183 cm^–1^, 156 × 193 cm^–1^. Cross-peaks not involving the ν(Ir–Ir) fundamental: 13 × 25, 11 × 50, 12 × 83, 7 × 250, 76 × 250, 82 × 50, 80 × 75, 198 × 91, 250 × 51, 289 × 249 cm^–1^, and the (150–200) × 249 cm^–1^ tail of the 132 × 242 cm^–1^ cross-peak. Additional vertical cross-peaks at 124 × 333 cm^–1^, 126 × 372 cm^–1^ (second overtone), 127 × 499 cm^–1^ (third overtone), 124 × 517, 126 × 530 cm^–1^ are in Figure S18B.

In the FSRS section, we presented oscillating intensities of individual FSRS features and determined the contributing frequencies by Fourier transformation (Figures S15–S17). Transforming the whole time-dependent FSRS in the 0–300 cm^–1^ range produced a 2D-FSRS map that provided a comprehensive picture of the vibrational coupling pattern averaged over the 4 ps time interval after actinic excitation. The map displays color-coded Fourier magnitudes as functions of frequencies of FSRS signals *and* frequencies of FSRS intensity oscillations (Figures 8 and S18; estimated peak positions stated in an FSRS × FT format). In other words, 2D-FSRS correlates exited-state vibrations activated by the joint action of Raman and probe pulses with Franck–Condon active vibrations that were coherently excited by the preceding 30 fs actinic pulse. Since FSRS were measured in the same spectral range as the FT oscillatory frequencies, 2D-FSRS of Ir(TMB) showed diagonal features that correspond to vibrations that were simultaneously Raman- and Franck–Condon active, as well as off-diagonal cross-peaks attributable^27,38,52,53^ to anharmonically coupled modes. 2D spectra detected modes whose Raman intensities oscillated with substantial amplitudes regardless their mean (nonoscillatory) values.

The strongest diagonal peak (128 × 124 cm^–1^) belonged to the ν(Ir–Ir) fundamental, followed by features due to the overtone at 247 × 241 cm^–1^, and deformation vibrations at 50 × 51 and 80 × 75 cm^–1^. The latter two were weaker in the 60-fs and absent in the 360-fs map (Figures 8 and S18D), likely because they originated from hot vibrations whose oscillations quickly diminished. Cross-peaks occurred between ν(Ir–Ir) and most of the vibrations in the investigated spectral range, producing horizontal off-diagonal features along the FT ν(Ir–Ir) wavenumber of about 125 cm^–1^ and vertical features in the 115–135 cm^–1^ range of periodically shifting ν(Ir–Ir) FSRS peak wavenumbers. Horizontal cross-peaks were stronger than their diagonal counterparts (or had no matching diagonal signals), suggesting that coupling with the ν(Ir–Ir) fundamental imparted FSRS intensity to the coupled modes whether they were actinically excited or not. Examples are the 16–18 cm^–1^ solute–solvent vibration whose cross-peak at 13 × 124 cm^–1^ was the strongest feature in the 2D spectrum, modes at ∼50 cm^–1^, between 70 and 100 cm^–1^ (apparent peak at 88 cm^–1^), the strong horizontal tail 140–200 cm^–1^, ν(Ir–Ir) overtone at 245 cm^–1^, and the 290 cm^–1^ cross-peak. Their Franck–Condon activities were assessed by inspecting vertical cross-peaks at the ν(Ir–Ir) FSRS position that correspond to actinically excited vibrations participating in the wavepacket and coupled with the ν(Ir–Ir) fundamental: the first, second, and third overtones (Figure S18B), features encompassed by the vertical tail below the ν(Ir–Ir) diagonal extending down to ∼40 cm^–1^, and a peak at 130 × 76 cm^–1^ plus weak features at 107 × 51, 126 × 25, and ca. 124 × 8 cm^–1^. Since these cross-peaks likely involve hot vibrations, they weakened and became fuzzy on going to the 60- and 360-fs map that skipped the initial dynamics. A branched vertical off-diagonal feature occurred in the 0-fs map just above the ν(Ir–Ir) diagonal, with weak cross-peaks at 114 × 191, 135 × 174, and 136 × 208 cm^–1^. In the 60-fs map, the two branches merged into a single tail with a peak at 122 × 194 cm^–1^ (Figure S18D) that moved in the 360-fs map to 127 × 183 cm^–1^ where it was accompanied by a new feature at 156 × 194 cm^–1^. In each map, vertical cross-peaks occurred across the full span of FSRS wavenumbers covered by the periodically shifting ν(Ir–Ir) FSRS peak (Figure 5), suggesting that the coupling strength depends on the wavepacket position on the Ir–Ir coordinate.

Strong horizontal off-diagonal feature at 140–200 cm^–1^ had neither diagonal nor vertical matching counterparts. The right-pointing strong horizontal tail of the ν(Ir–Ir) peak at (140–200) × 125 cm^–1^ is not a cross-peak but a 2D manifestation of the broad minimum on the high-wavenumber side of the ν(Ir–Ir) FSRS peak. As an inherent part of the dispersive-shaped ν(Ir–Ir) band, it oscillated with the ν(Ir–Ir) frequency of about 125 cm^–1^ instead of the frequencies in its own range, 140–200 cm^–1^. The ν(Ir–Ir)–overtone vertical cross-peak at 132 × 242 cm^–1^ mirrored the full shape of the ν(Ir–Ir) diagonal feature, including the high-wavenumber tail. The 290 × 124 cm^–1^ feature may be due to a mode that was not actinically activated but acquired some FSRS intensity through coupling with ν(Ir–Ir).

The 65 cm^–1^ band (from late-time FSRS) was not observed either on or off the diagonal, owing to its slow formation during vibrational relaxation (it gained intensity late, when oscillation amplitudes were very small: a faint diagonal feature emerged at 60 cm^–1^ in the 360-fs map.) The strongest FSRS feature at 16–18 cm^–1^ was very weak at the diagonal but its cross-peak with ν(Ir–Ir) was the most intense feature in the 2D spectrum (Figure S18A); and it also produced cross-peaks with virtually all other detected vibrations: 25, 51, 83, 250 cm^–1^, and possibly 158, 182, and 208 cm^–1^ as well.

To summarize, 2D-FSRS demonstrated a network of coupled low-frequency vibrations that were coherently excited by the actinic pulse and formed a wavepacket consisting predominantly of higher ν(Ir–Ir) levels, plus weaker contributions from the first (second, third) overtones and hot deformation modes at 50 and 75–80 cm^–1^. Coupling with the high-intensity ν(Ir–Ir) mode enhanced FSRS features due to these as well as some other modes, namely the solute–solvent vibration at 16–18 cm^–1^. Some of the modes were activated through coupling with ν(Ir–Ir) and participated in the wavepacket even if virtually not excited actinically and very weakly FSRS active (25, 174, 184, 208 cm^–1^).

### Phototriggered Ir–Ir Bond Formation

The ^1^dσ*pσ excited state of Ir(TMB) was structurally characterized by late-time (>5 ps) SE and FSRS spectra, as well as TDDFT. The excited-state Ir–Ir interaction is much stronger than that in the ground state, with the ν(Ir–Ir) wavenumber increasing from 53 cm^–1^ in the GS^28^ to 126 cm^–1^ in the excited state (the calculated bond order increases from 0.06 to 0.35 and the Ir–Ir distance shortens by 0.28 Å). TDDFT, which predicted that the ^1^dσ*pσ PES is anharmonic along the Ir–Ir coordinate, also found four different conformers whose excited-state energies become nearly degenerate at shorter Ir–Ir distances (Figure 3 and Table S2). Two bands in the late-time SE spectrum, SE_L_ and SE_H_ in a 1:4 area ratio, are attributable to two conformers (Figure S2A), but FSRS showed only a single ν(Ir–Ir) band at 126 cm^–1^ (Figure 5B). Greater strengthening of the metal–metal interaction upon excitation, PES anharmonicity, and degree of conformational flexibility are main distinctions between Ir(TMB) and Pt(pop), the prototypical d^8^–d^8^ complex whose Pt–Pt stretching frequency increases upon ^1^dσ* → pσ excitation from 119 to 149 cm^–1^ and its excited-state PES is harmonic up to high vibrational levels.^10,11,54^

We turn now to ultrafast time-resolved experiments that revealed phototriggered Ir–Ir bond formation in the ^1^dσ*pσ excited state to be a complex dynamical process. Oscillating SE and FSRS intensities and their Fourier spectra are clear evidence for the formation of a vibrational wavepacket mainly made up of ν(Ir–Ir) with smaller contributions from deformation vibrations. SE spectral shifts were consistent with a time-dependent energy gap and Franck–Condon overlap between the excited- and ground states while FSRS revealed periodic structural changes and involvement of hot vibrations. Nonsinusoidal periodic time evolutions of both FSRS and SE spectral patterns pointed to wavepacket movement on an anharmonic/multidimensional ^1^dσ*pσ PES, where the principal motion along the ν(Ir–Ir) coordinate is accompanied by small conformational changes whereby the wavepacket returned by a different path. Finally, 2D-FSRS revealed extensive coupling between ν(Ir–Ir) and deformation modes.

These observations led to the following model (Figure 3). Actinic excitation generated a distribution of higher ν(Ir–Ir) vibrational levels with a maximum around *v* = 10 (Table S6) as well as hot deformation vibrations. The wavepacket was established in a few tens of femtoseconds while moving toward shorter Ir–Ir distances. (SE and FSRS signals were faint in this period. Eventually, LE-SE and the ν(Ir–Ir) FSRS peak emerged, marking the beginning of well-observable periodic changes.) The wavepacket moved from the right (long Ir–Ir) turning point toward the short Ir–Ir region where the PES became steeper and rugged, owing to near-degeneracy of conformer energies. As the ν(Ir–Ir) wavenumber increased to 136 cm^–1^ (140 cm^–1^ in Stokes), the wavepacket turned back, transiently visiting a region above a local minimum of another conformer (HE-SE diminishing, ν(Ir–Ir) downshifting) and finally rapidly returned toward the right turning point where ν(Ir–Ir) dropped to about 118 cm^–1^ (105 cm^–1^ in Stokes) and then began increasing again together with FSRS and LE-SE intensities at the beginning of the next period. This ∼270 fs cyclical process also involved changes in deformation-mode populations. It was accompanied by 1–1.5 ps vibrational relaxation whereby the wavepacket moved on progressively narrower loops spanning a smaller Ir–Ir distance range, eventually splitting and relaxing into two local minima manifested by the SE_H_ and SE_L_ features in late-time spectra.

It can be concluded that Ir–Ir bonding arises upon Franck–Condon excitation whereby the ν(Ir–Ir) peak jumps from 53 to 124 cm^–1^. Adjustment of dihedral angles and ligand periphery structure is aided by simultaneous activation of coupled deformation modes. Formation of equilibrated excited-state Ir–Ir bond vibrating at 126 cm^–1^ then proceeds through complicated wavepacket motions on a multidimensional ^1^dσ*pσ PES involving periodic bond weakening/strengthening, transient conformational changes enabled by a network of coupled hot deformation vibrations, and simultaneous descent to energetic minima belonging to two conformers.

Of interest is that relaxation and decoherence kinetics of Ir(TMB) were almost the same in THF, AN, and BN, especially in view of vastly different solvation times of the latter two solvents. This finding is in stark contrast to 0.5 ps desolvation and following 3 ps reorientation/resolvation indicated by ultrafast diffuse X-ray scattering^15^ for the long-isomer of Ir(dimen). The different behavior could be due to the much larger Ir–Ir contraction in Ir(dimen) than in Ir(TMB) (1.4 vs 0.28 Å), as there would be much greater perturbation of the Ir(dimen) solvation shell.

Unusually large oscillation amplitudes are another intriguing aspect of Ir(TMB) photophysics. Amplitudes of SE and FSRS intensity oscillations in the first 1–2 ps made up the signals instead of just modulating them as is the case for other systems in solution (for example, TA spectra of d^8^–d^8^ complexes Pt(pop), Ir(dimen), and [Pt_2_(μ-L)_2_(N^∧^C)_2_]). The exceptional activity of Ir(TMB) is attributable to large displacements and very different shapes of the ground- and excited-state PESs. The wavepacket moves over a large area where the GS energy gap and Franck–Condon overlap strongly depend on its position. SE then oscillates with huge amplitudes, carrying FSRS signals along through resonance enhancement. The estimated decoherence time was comparable to the vibrational relaxation time (1–1.5 ps), which implies that vibrational dephasing is slower than decay of individual higher vibrational levels and that dephasing occurs through intramolecular vibrational energy dissipation to other modes.^10,55^ This interpretation is supported by 2D-FSRS evidence for an extensive network of coupled low-wavenumber hot vibrations and by the lack of dynamic solvent effects. In vibrational dephasing, Ir(TMB) behaves more like Pt(pop)^10,11^ than Ir(dimen), whose coherence loss has been attributed^56^ to a statistical mechanism in a large spread of initial structures.

Employing optically induced coherence to facilitate photophysical/photochemical processes is a topic of interest with important implications for photocatalysis, light-energy conversion and storage, as well as photobiology.^57−60^ For example, a ν(Pt–Pt) wavepacket in [Pt_2_(μ-L)_2_(N^∧^C)_2_] complexes periodically changes energy differences between the lowest excited singlet and intermediate states, accelerating intersystem crossing (ISC) to the lowest triplet.^16^ This is not the case in the photophysics of Ir(TMB), where the lowest singlet and triplet dσ*pσ states are energetically isolated and ISC is much slower (70 ps),^3^ unaffected by coherent oscillations. On the bright side, studying Ir(TMB) vibrational coherence shed new light on excited-state bond formation dynamics that could not be easily extracted from most other experiments.

2D-FSRS constructed from spectra measured in the range of signal oscillation frequencies turned out to be useful in directly identifying excited and coupled vibrations and revealing their roles in excited-state evolution. This approach could shed light on a variety of other photo(catalytically) active binuclear systems^61−65^ and molecular metal clusters with active low-frequency modes.

## Conclusions

Using time-resolved stimulated emission and femtosecond stimulated Raman spectroscopy (FSRS) in the low-frequency range of skeletal vibrations, we followed metal–metal bond formation upon 620-nm dσ* → pσ excitation of a di-iridium(I) isocyanide complex. 30-fs excitation triggered exceptionally large coherent oscillations of spectroscopic signals whose Fourier transforms revealed optically activated Ir–Ir stretching vibrations and, to a lesser extent, ligand deformations and torsional motions. Excited-state vibrational modes were directly observed in impulsive stimulated Raman spectra (ISRS) as well as in FSRS that showed their dynamic evolution. In both experiments, excited-state Raman signals were enhanced by resonance with stimulated emission.

An ″instantaneous″ increase of the Ir–Ir stretching frequency in FSRS and its subsequent nonsinusoidal dynamic shifts in the 105–140 cm^–1^ range confirmed that the Ir–Ir bond forms impulsively and then evolves through periodically repeating intervals of rapid weakening and slower strengthening that last for about 50 and 220 fs, respectively. Simultaneously occurring vibrational relaxation diminishes oscillation amplitudes and eventually produces two relaxed excited-state conformers in about a 4:1 ratio. Conformers were distinguishable in the stimulated emission spectrum while showing a common ν(Ir–Ir) FSRS peak at 126 cm^–1^, which is double the ground-state value. Relaxation and decoherence occurred with comparable lifetimes of 1–1.5 ps. Excited-state dynamics were accounted for by damped looplike wavepacket motion on a multidimensional anharmonic potential energy surface involving cycles of Ir–Ir bond weakening/strengthening, transient conformational changes enabled by coupled hot deformation modes, and simultaneous branched relaxation.

A 2D-FSRS map (constructed by combining Fourier frequency spectra of coherent Raman intensity oscillations and FSRS measured in the same low-frequency range) distinguished Franck–Condon activated modes from those gaining Raman intensity through coupling with the Ir–Ir stretch. The map revealed a network of coupled vibrations that facilitates structural relaxation accompanying periodic changes in the Ir–Ir distance. Coherent vibrational motions and coupled vibrations did not accelerate intersystem crossing that was much slower (70 ps) than vibrational dephasing.

Our findings demonstrate the power of time-resolved excited-state Raman spectroscopy and its 2D extension to identify activated skeletal vibrations, their anharmonic couplings, and unravel ultrafast structural changes brought about by electronic excitation.

## Data Availability

Data are openly available from ZENODO at DOI 10.5281/zenodo.14636984 or on request.

## Supporting Information Available

Additional calculated structural and vibrational data, characterization of GS and excited conformers; TA and Raman spectra (FSRS, ISRS, 2D) and Fourier frequency spectra of SE and FSRS signal oscillations; experimental section: sample synthesis and handling; description of FSRS and ISRS setups; details of TDDFT calculations; and wavepacket simulations (PDF)
