# Supplementary material for: Real-Time Tracking of Photoinduced Metal–Metal Bond Formation in a d8d8 Di-Iridium Complex by Vibrational Coherence and Femtosecond Stimulated Raman Spectroscopy
Source: J Am Chem Soc. 2025 Mar 6;147(11):9810–24. doi: 10.1021/jacs.4c18527 (PMC11926863; doi:10.1021/jacs.4c18527)
Supplement: Supplementary file 1 — ja4c18527_si_001.pdf [file ja4c18527_si_001.pdf]

SUPPORTING INFORMATION

Real-time Tracking of Photoinduced Metal-Metal Bond Formation in a d^8d^8 Di-Iridium Complex by Vibrational Coherence and Femtosecond Stimulated Raman Spectroscopy

Miroslav Kloz,*[,a] Jakub Dostál,[a] Atripan Mukherjee,[a] Martin Pižl,[b] Filip Šebesta,[c,d] Michael G. Hill,[e] Harry B. Gray,[f] Stanislav Záliš,[d] Antonín Vlček*[,d,g]

[a] Extreme Light Infrastructure ERIC, ELI Beamlines Facility, Za Radnicí 835, 252 41 Dolní Břežany, Czech Republic
[b] Department of Inorganic Chemistry, University of Chemistry and Technology Prague, Technická 5, CZ-166 28 Prague, Czech Republic
[c] Department of Chemical Physics and Optics, Faculty of Mathematics and Physics, Charles University, Ke Karlovu 3, CZ-121 16 Prague, Czech Republic
[d] J. Heyrovský Institute of Physical Chemistry, Czech Academy of Sciences, Dolejškova 3, CZ-182 23 Prague, Czech Republic
[e] Department of Chemistry, Occidental College, Los Angeles, CA90041, USA
[f] Beckman Institute, California Institute of Technology, Pasadena, California 91125, United States
[g] Department of Chemistry, Queen Mary University of London, E1 4NS London, U.K.

Table of Contents

Figures and tables:

Calculated structural and spectroscopic data, conformers characterization	S3-5
UV-vis absorption spectra	S6
Stimulated emission	S7-12
Calculated vibrations	S13-16
Wavepacket movement	S17
ISRS	S18
FSRS in AN	S19-26
2D-FSRS	S27
FSRS in BN and THF	S28-29

Experimental

Sample synthesis and handling	S30
FSRS	S30-32
ISRS	S32-33
Quantum chemical calculations	S33-34
Wavepacket simulations	S34-36

References	S37-38

Table S1. Ir(TMB) structural, IR and Raman spectroscopic data.

	**GS**	**^{1}dσ*pσ**	**^{3}dσ*pσ**
Ir-Ir (Å) [a]	3.16 (exp 3.12)[1]	2.87	2.85
C-Ir-Ir-C (°) [a]	27.7 (exp 27)[1]	27.9	28.0
ν(Ir-Ir) (cm^{-1}) exp.	53 [1]	122-126 [b]	132 [1]
ν(C≡N) (cm^{-1}) exp., IR[2]	2147 (w), 2158 (s), 2203 (w)	2140	2148
Ir–Ir bond order[2]	0.062	0.346	0.416
C≡N bond order[2]	2.635	2.609	2.388

[a] DFT, this work, [b] FSRS at time delays >4 ps, this work.

Table S2. DFT-calculated free energies (eV) relative to the most stable form and Ir-Ir distances (Å) of Ir(TMB) conformers in the ground and $^{1}d\sigma^{*}p\sigma$ states in AN. Structures are shown in the last two columns. The conformer notation specifies the numbers of TMB ligands adopting the same local structure (ligands folded as in the lowest GS form: ligands with switched C–C orientation). The bond(s) switching orientation is (are) highlighted.

	GS		$^{1}d\sigma^{*}p\sigma$			
**Conformation**	**ΔG**	**r(Ir–Ir)**	**ΔG**	**r(Ir–Ir)**	**Structure projected along the M–M axis**	**Structure projected in the direction perpendicular to the M–M axis**
4:0<br>D_4 symmetry<br>Lowest GS energy	0.000	3.160	0.071	2.874	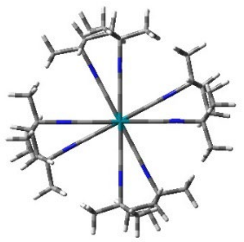	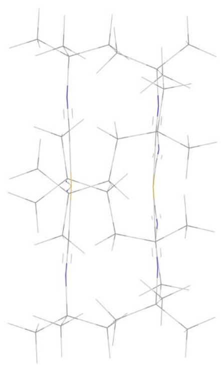
3:1	0.148	3.155	0.000	2.946	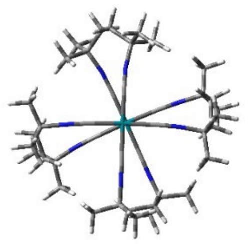	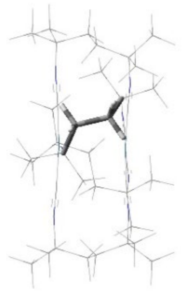
trans-2:2	0.234	3.111	0.043	2.919	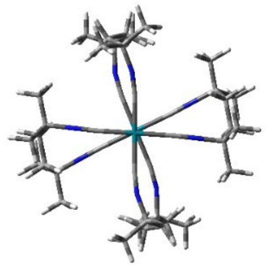	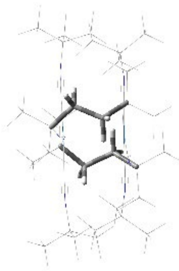

cis-2:2	0.168	3.129	0.036	2.946	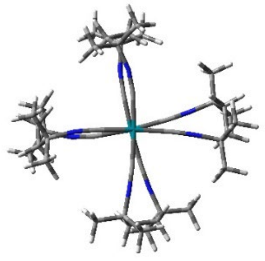	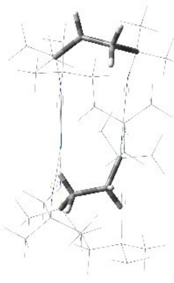

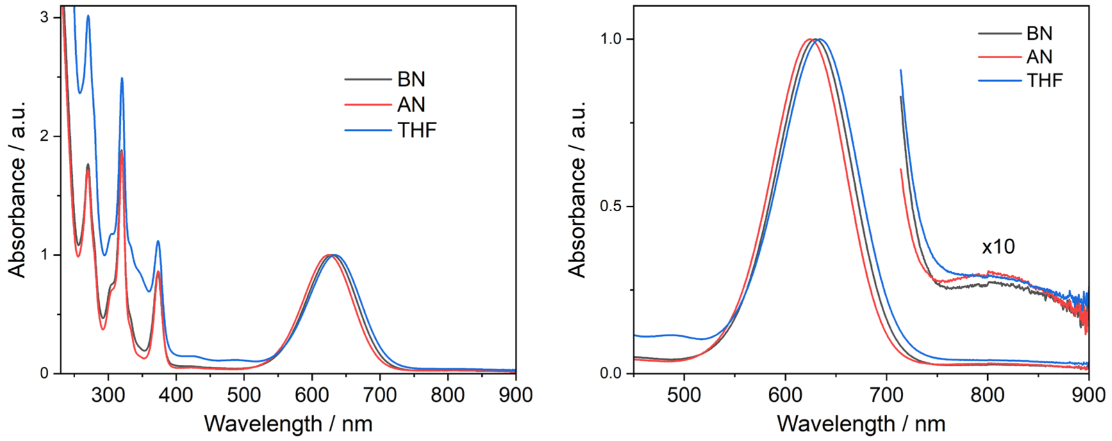

Figure S1. UV-vis absorption spectra of Ir(TMB) in three solvents normalized at ~630 nm. Left: full range. Right: detail of the $^{1}d\sigma^{*}\rightarrow p\sigma$ absorption. The $^{1}d\sigma^{*}\rightarrow p\sigma$ peak position is slightly solvent-dependent: 630 nm (BN), 625 nm (AN, ε = 11,200 $M^{-1}cm^{-1}$),[3] 634 nm (THF). A weak feature around 800 nm corresponds to the spin-forbidden $^{3}d\sigma^{*}\rightarrow p\sigma$ transition. For a full spectral assignment, see refs.[1-3] Solvent abbreviations: BN = butyronitrile, AN = acetonitrile, THF = tetrahydrofuran.

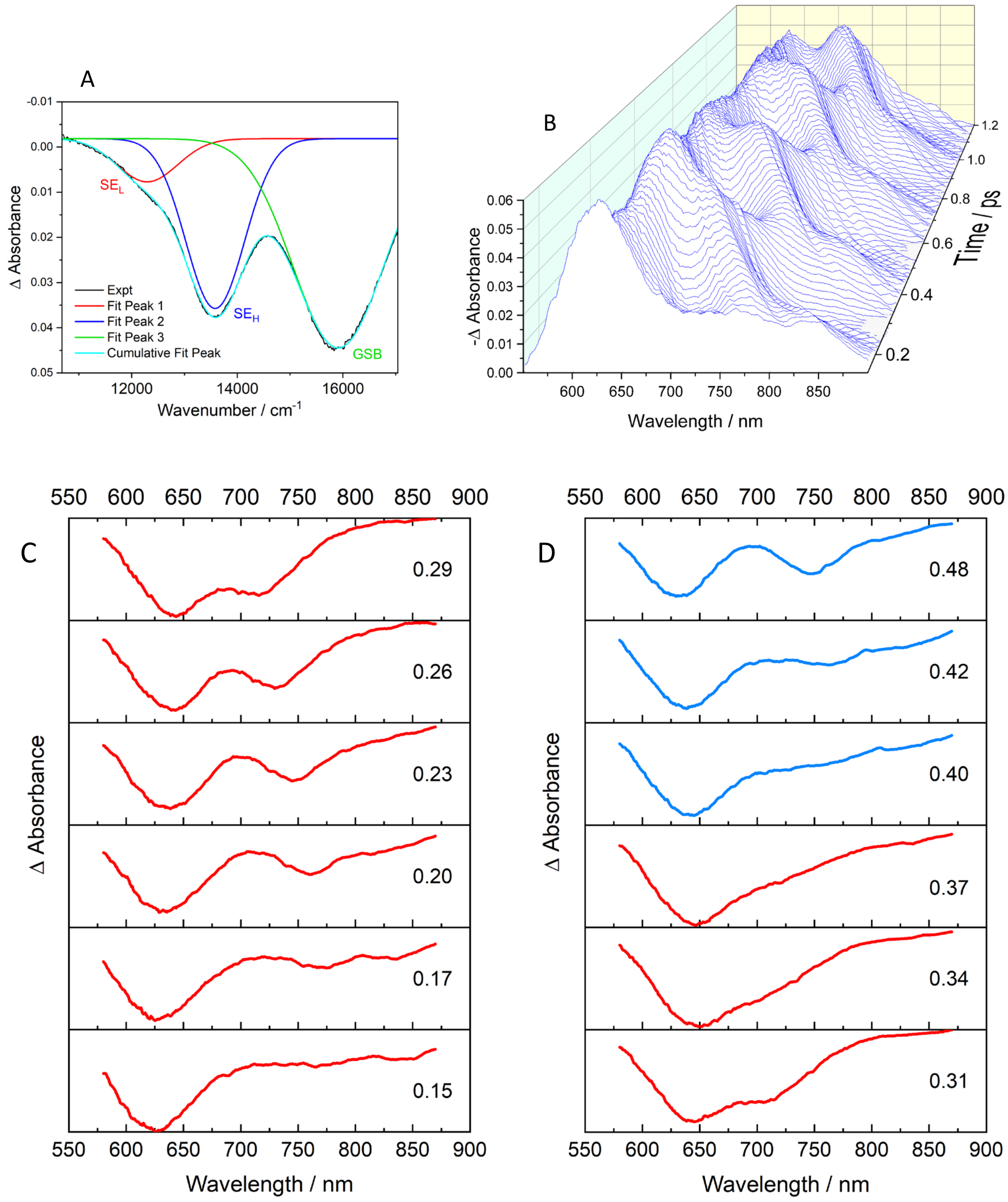

Figure S2. A: Transient absorption spectrum of Ir(TMB) in BN measured 19.46 ps after excitation, its Gaussian decomposition to the GSB band at 15910 cm^{-1} (629 nm), and two SE bands at 13580 cm^{-1} (736 nm, SE_H) and 12300 cm^{-1} (813 nm, SE_L). (Subscripts H, L denote high- and low-energy.) SE_H/SE_L band-area ratio = 3.7.

B: "Waterfall" representation of TA spectra measured 0.14-1.2 ps after excitation. Plotted with reversed sign (-Δ Abs) to show SE and GSB as positive maxima. GSB appears on the left. Blue-shifting and rising HE-SE appear as "ridges" increasing leftwards and backwards toward apparent

maxima in the central part. LE-SE appears as lower "ridges" on the right. Regions of strong SE are separated by deep valleys. Shallow saddles connecting the ridges originate from the ν(Ir-Ir) overtone. They match the white-pink stretches connecting SE regions in the time × wavelength map (Figures 1-C and S3).

C, D: Selected spectra of the first full oscillation period and at the beginning of the second. Signal-intensity (Δ Absorbance) ranges -0.06 (bottom) - 0 (top) in all C panels and -0.068 - 0 in D. SE starts as a broad signal between 900 and 680 nm, gradually shifts to shorter wavelengths and diminishes in intensity in the red part of the spectrum. A distinct HE-SE peak emerges at ~760 nm (0.17 ps), shifts to shorter wavelengths and overlaps with the GSB at 0.34-0.37 ps when it vanishes. The broad LE-SE signal re-emerges at the beginning of the second oscillation 0.37-0.4 ps (the first blue curve). The HE-SE starts developing again into a distinct peak at about 760 nm (0.42 ps). Then it grows and shifts to shorter wavelengths while LE-SE decreases. The same behavior repeats itself with diminishing amplitudes until ca. 4 ps. The signal in the "saddle" region (ca. 725-760 nm) oscillates alongside the HE-SE but the intensity drops less in the minima. Eventually, the SE intensity, which concentrates in this region, evolves into the SE_H band seen at long time delays.

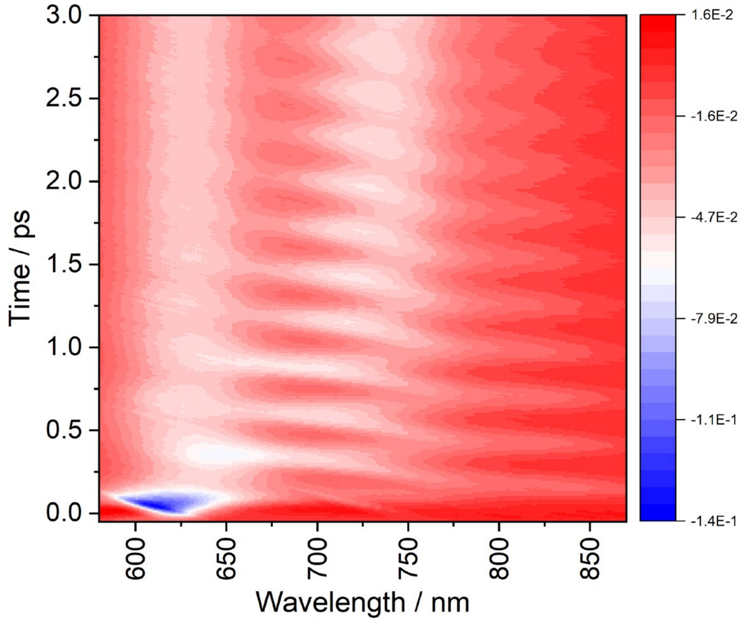

Figure S3. Time × wavelength map showing periodic evolution of the GSB/SE spectral pattern over a 0.05 – 3.0 ps range. Blue/white colors correspond to the strongest SE signal and minima are in red. The HE-SE peak shifts toward longer wavelengths with successive oscillation periods and spectrally narrows owing to vibrational relaxation.

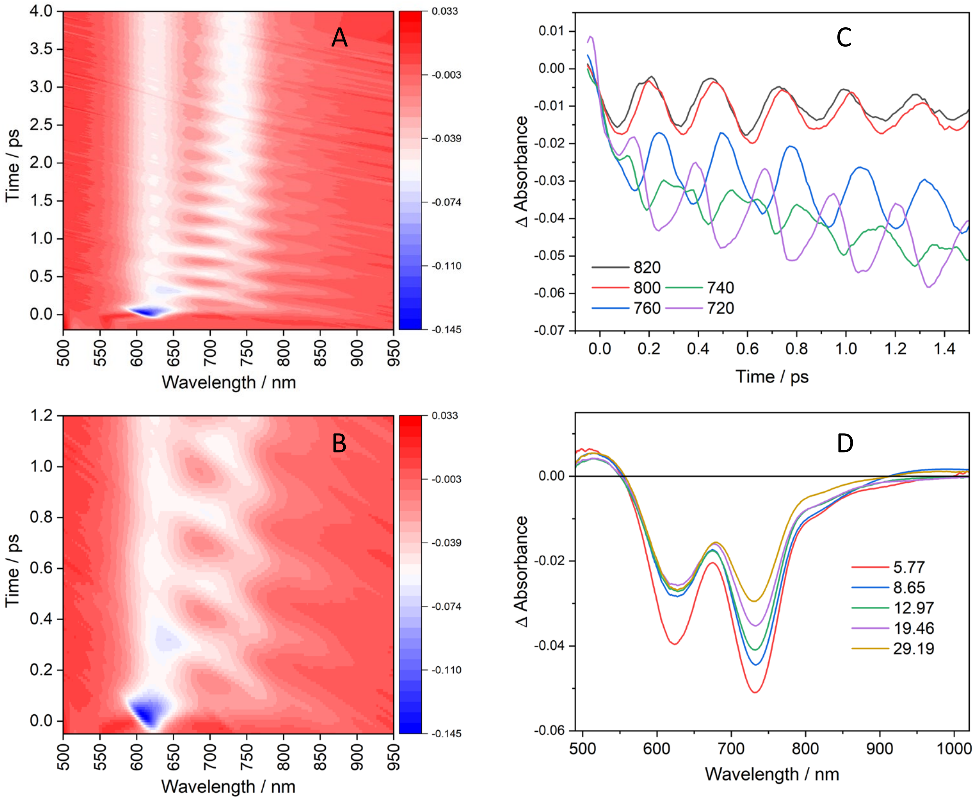

Figure S4. Overview of Ir(TMB) TA spectra in AN. A: Time × wavelength map showing periodic evolution of the GSB/SE spectral pattern over a -0.2 – 4.0 ps range. Blue/white colors correspond to the strongest SE signal, minima in red. B: Detail of A showing time evolution up to 1.2 ps. C: Time profiles at selected detection wavelengths. D: Late-time spectra measured at selected time delays (ps).

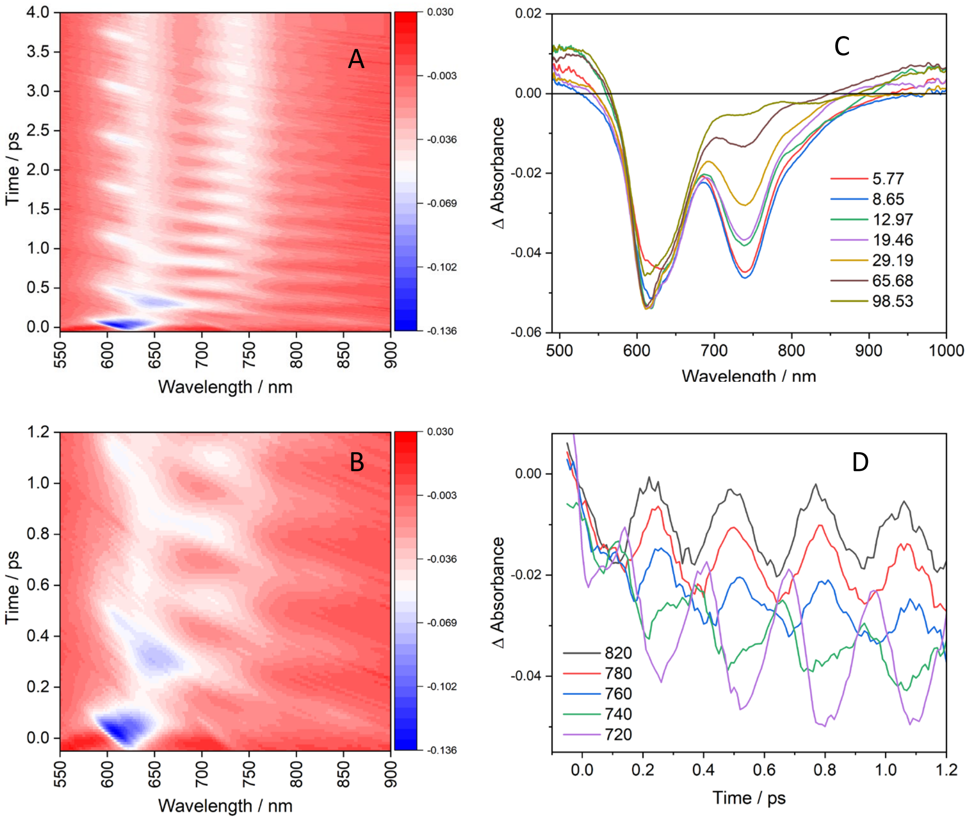

Figure S5. Overview of Ir(TMB) TA spectra in THF. A: Time × wavelength map showing periodic evolution of the GSB/SE spectral pattern over a -0.2 – 4.0 ps range. Blue/white colors correspond to the strongest SE signal, minima in red. (Note the pronounced low-frequency oscillations on the blue GSB side due to GS ν(Ir-Ir) vibrations. B: Detail of A showing time evolution up to 1.2 ps. C: Time profiles at selected detection wavelengths. D: Late-time spectra measured at selected time delays (ps).

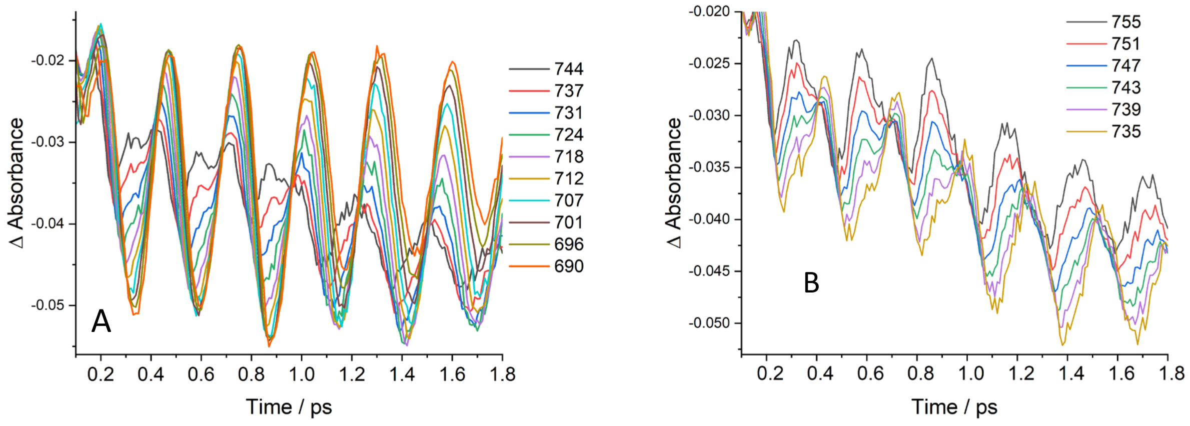

Figure S6. Oscillating SE intensities of Ir(TMB) in BN measured at selected chirp-corrected detection wavelengths in 10 fs steps. A: Time-profiles measured across the short-wavelength region showing oscillations shifting later in time with decreasing detection wavelength, owing to the SE dynamic blue shift. Contribution from the overtone frequency is apparent in the middle of oscillation minima. B: detail of the phase-shift region.

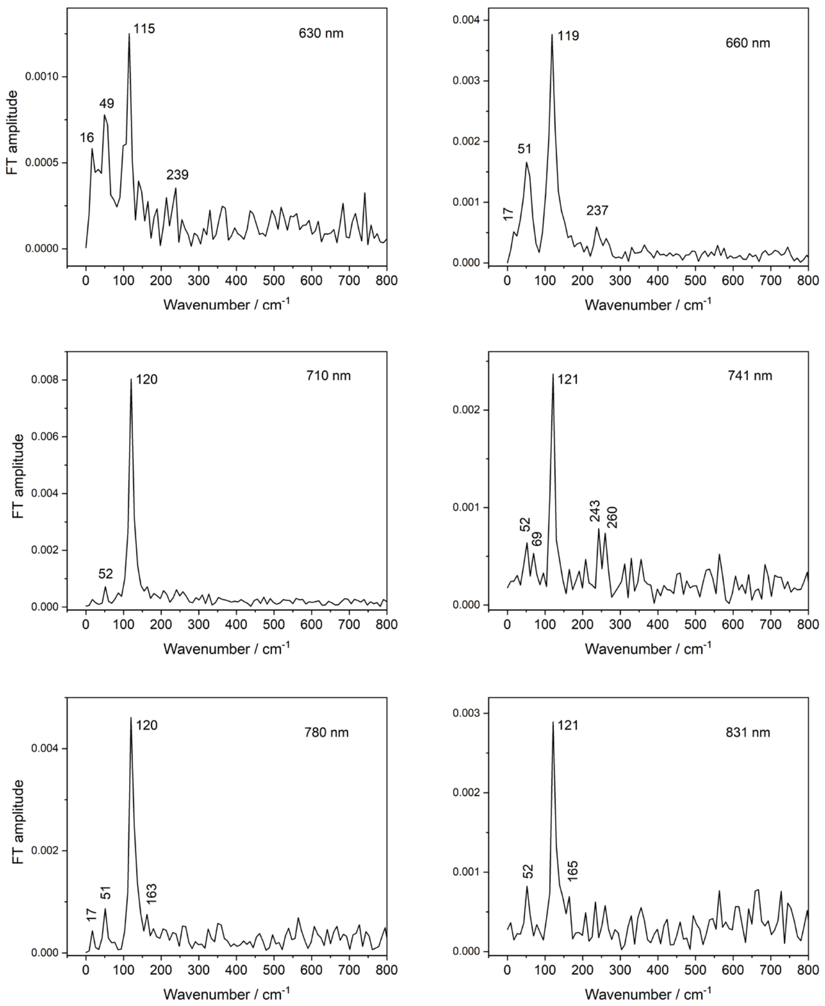

Figure S7. FT spectra of oscillating SE intensity time profiles measured at selected detection wavelengths in AN.

Table S3. Selected TDDFT-calculated harmonic vibrations of the Ir(TMB) $^1d\sigma^*p\sigma$ excited state in AN. Wavenumbers and intensities were calculated without any symmetry constraints. Wavenumbers were scaled by a factor of 0.956. Raman scattering activities do not account for resonance enhancement and, therefore, cannot be related to the present experiments. Symmetry representations were determined by a separate calculation assuming idealized D_4 molecular symmetry. Totally symmetric vibrations are highlighted in yellow. ν(Ir-Ir), ν(C≡N) = stretching vibrations of Ir–Ir and C≡N bonds. Torsion = rotation of the Ir(C≡N–)$_4$ planes around the Ir–Ir axis in opposite directions, periphery = deformation motions of the complex periphery, mainly involving TMB C–H bonds, breathing: expansion/contraction perpendicular to Ir–Ir, δN = bending at N atoms in Ir(C≡N–)$_4$ planes, δC = bending at C atoms in Ir(C≡N–)$_4$ planes. Vibrational motions are shown in Figure S8.

**Wavenumber** cm^{-1}	**Symmetry**	**R. act.** Å^4/AMU	**Description**	**IR int.** km/mole
29	B_1	2	torsion, periphery, δN	
39	B_2	7	torsion, periphery, δN	
51	B_2	4	torsion, periphery	
52	A_1	0.1	dihedral, periphery	
75	A_1	4	torsion, periphery, δN	
90	B_2	7	torsion, δ(N), periphery	
94	A_1	0.5	dihedral, periphery	
118	B_1	6	dihedral, δ(N), periphery	
**122**	**A_1**	**16**	ν(Ir-Ir), δC, δN periphery	
131	B_2	2	periphery	
158	B_2	10	periphery, δ(N)	
185	B_1	6	periphery-antisymm. breathing	
187	A_1	16	periphery-breathing, δN, δC	
221	A_1	10	periphery-breathing, δN	
237	A_1	2	periphery	
250	E	4		
260	A_1	3		
281	A_1	3	periphery, δC	
316	E	50		
354	E	52		
445	A_1	17		
485	E	33	periphery, δC	
517	A_1	8	periphery, ν(Ir-Ir), δC, δN	
544	B_2	10		
551	B_1	6		
**ν(C≡N) vibrations**				
2153	E	228		374
2155	B_2	2668		0

2157	B_1	10873		0
2158	E	8		11789
2206	A_2	1		6
2235	A_1	7655		0

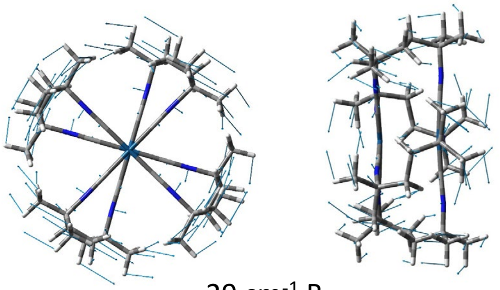

29 cm^{-1} B_1

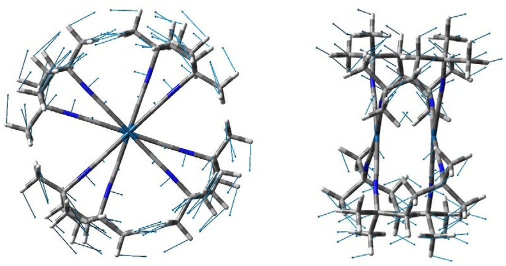

39 cm^{-1} B_2

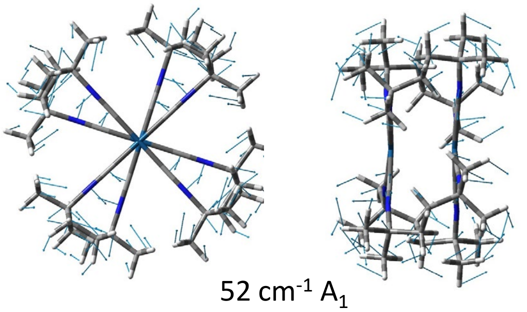

52 cm^{-1} A_1

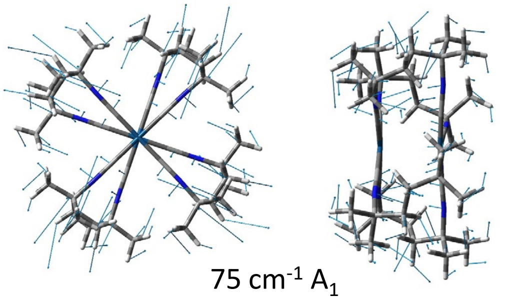

75 cm^{-1} A_1

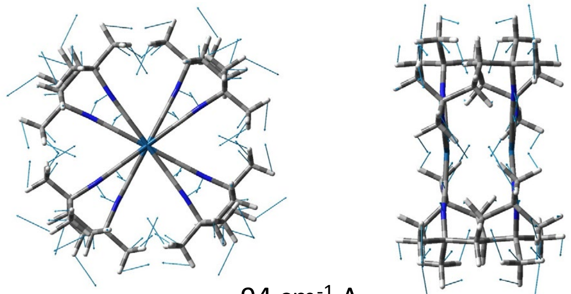

94 cm^{-1} A_1

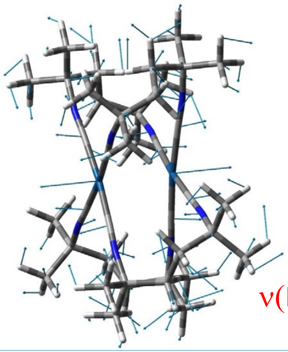

ν(Ir-Ir) 122 cm^{-1} A_1

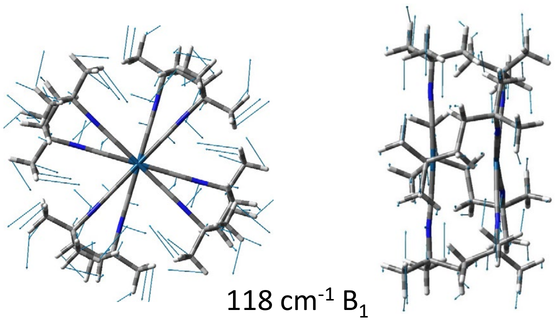

118 cm^{-1} B_1

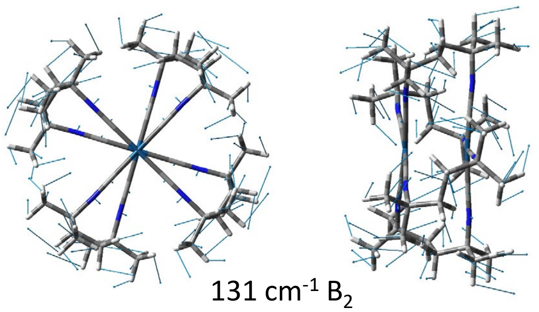

131 cm^{-1} B_2

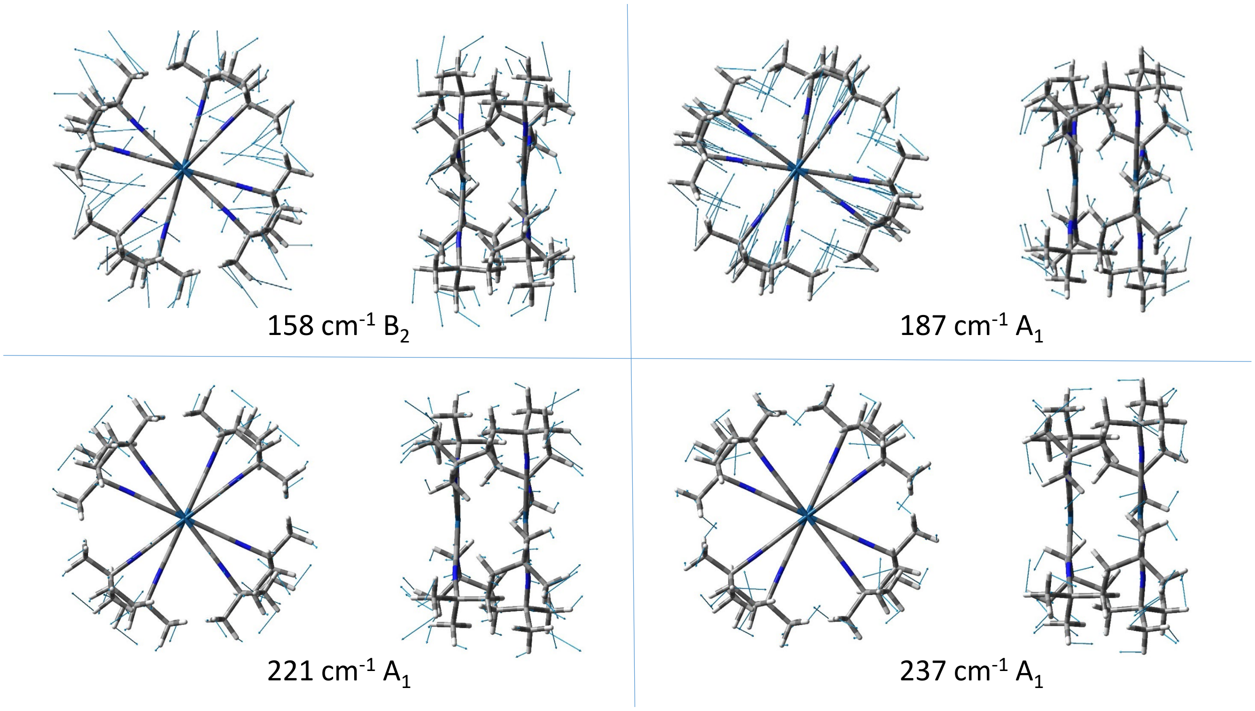

Figure S8. Calculated harmonic vibrations of the Ir(TMB) ^{1}dσ*pσ state in AN. Wavenumber values were scaled by 0.956.

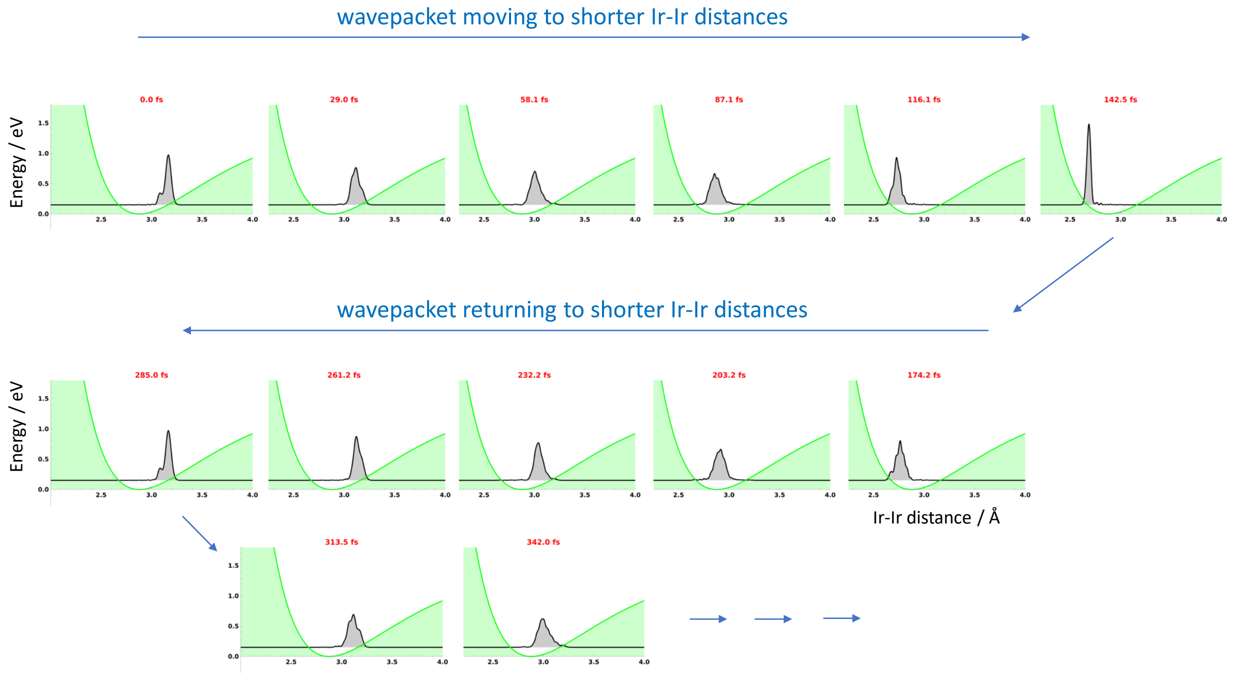

Figure S9. Simulated ν(Ir-Ir) wavepacket movement on TDDFT–calculated PES of the Ir(TMB) $^{1}d\sigma^{*}p\sigma$ excited state. The simulation started at the point of Franck-Condon excitation from the ground state (0.0 fs). Upper row: the wavepacket moves to shorter Ir-Ir distances and reaches the left (short-distance) turning point (142.5 fs). Lower row: the wavepacket moves to longer distances and returns to the FC point (285.0), then starts moving left again (shown at 313.5, 342.0, … fs). Details of the simulation, FC overlaps, and energies of ν(Ir-Ir) levels are summarized in Experimental section, p. S35. The simulation shows that: the wavepacket has a different shape at the left and right turning points; the wavepacket is broader in the region between the turning points; and the wavepacket shape at a given Ir-Ir distance is independent of its history – whether the wavepacket arrived from the left or right.

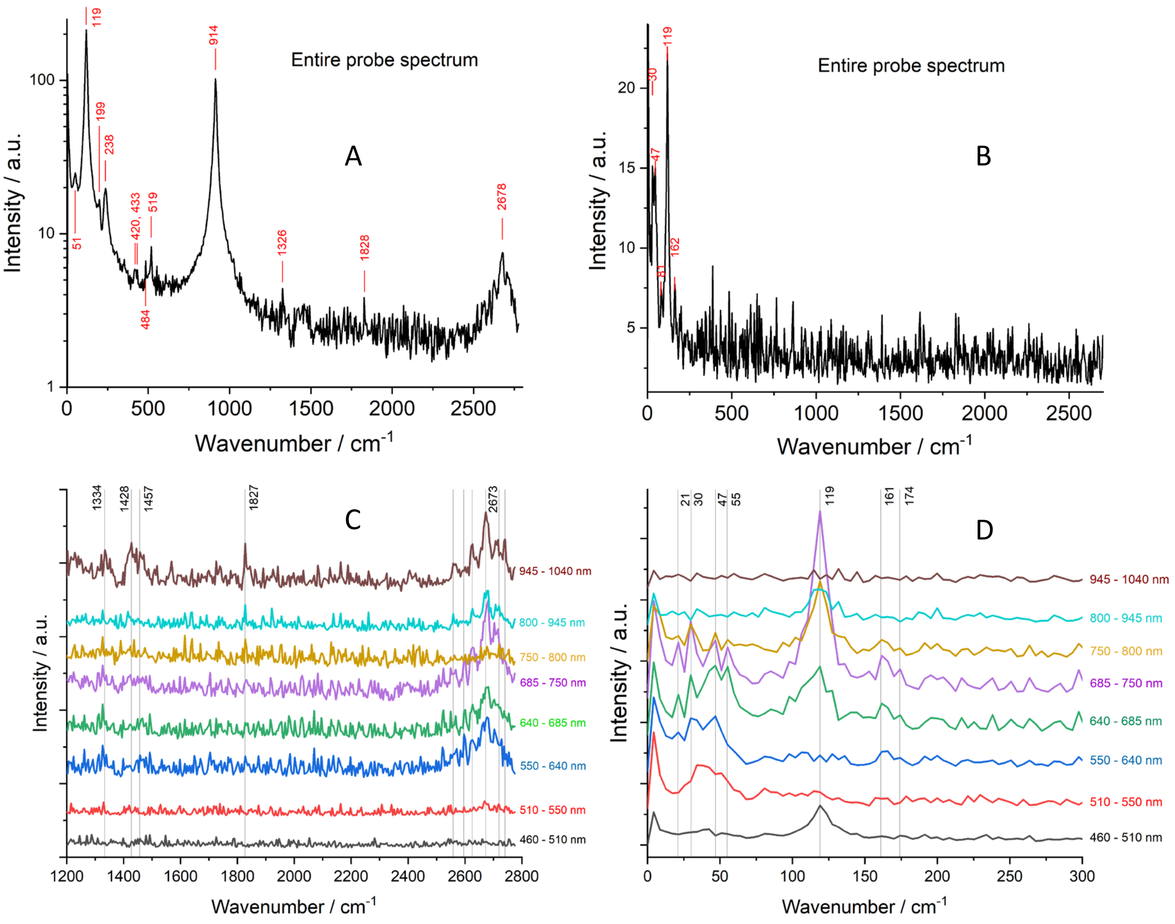

Figure S10. Ir(TMB) ISRS in THF solution. A, B: spectra obtained by FT of the whole probe wavelength range without (A, log intensity scale) and with (B) the actinic pre-pulse. C: ISRS recorded in the high-frequency range without the pre-pulse. FT performed in selected probe wavelength ranges. Peaks at/around 2673 cm^{-1} are aliased due to undersampling. Their actual wavenumbers equal 5550 – ν, where ν is the wavenumber of the aliased band. The center peak corresponds to 2877 cm^{-1}, attributable to C–H vibrations. D: ISRS recorded over different probe-wavelength intervals 300 fs after the actinic pre-pulse. The experiment was run in THF to keep the ν(C≡N) region free of solvent signals. None of the spectra in panels A, B, C shows any signal attributable to ν(C≡N) vibrations, see Table S2 for expected wavenumbers. A strong THF peak occurred at 914 cm^{-1}.

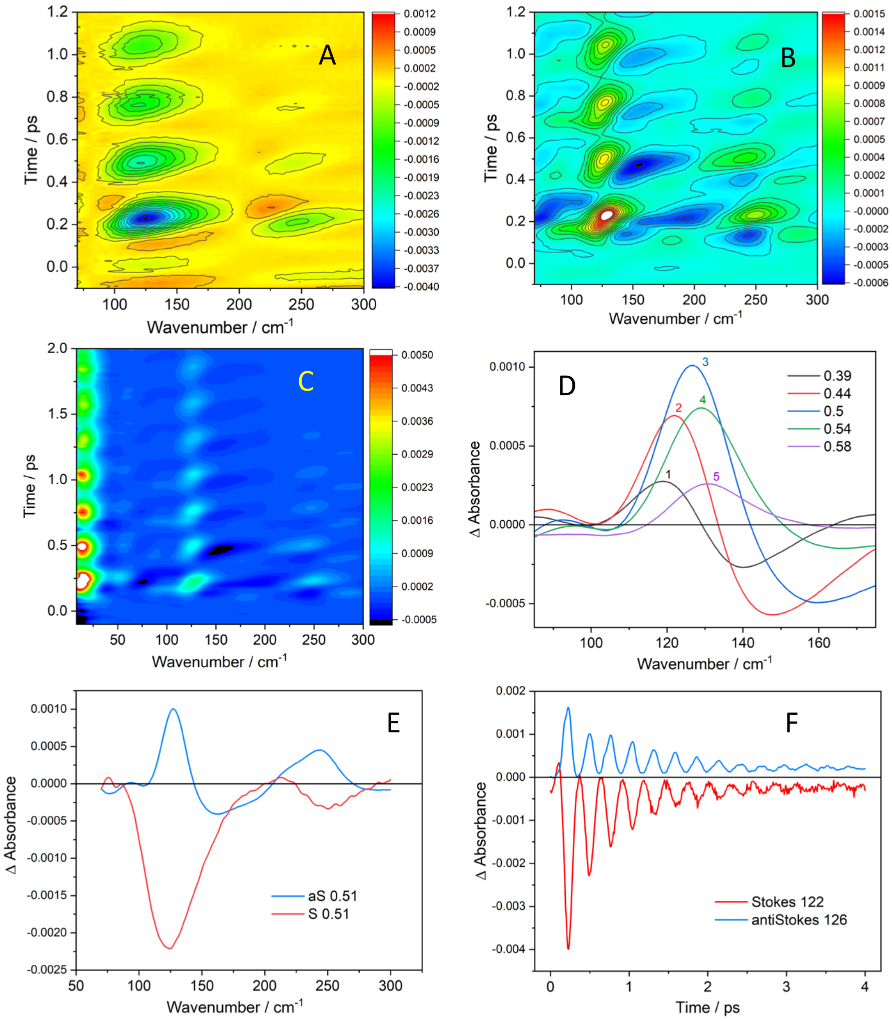

Figure S11. Details of excited-state FSRS in AN. A, B: comparison of Stokes (A) and anti-Stokes (B) spectra recorded over the first 1.2 ps after excitation. C: wavenumber × time map of anti-Stokes FSRS. Scale adjusted to visualize the 16-18 cm^{-1} feature. D: shifts and shape changes of the ν(Ir-Ir) band during the second oscillation period. E: Stokes (red) and anti-Stokes (blue) FSRS measured at 0.51 ps. Broad negative Stokes ν(Ir-Ir) band at 124 cm^{-1} encompasses the anti-Stokes 94 cm^{-1} shoulder and the 127 cm^{-1} maximum together with the 163 cm^{-1} negative satellite. The weak feature at 76 cm^{-1} has opposite sign in the two spectra. The 210-220 cm^{-1} feature appears as a positive shoulder in anti-Stokes and weak positive Stokes band at 212 cm^{-1}. The overtone occurs as a broad positive anti-Stokes band at 244 cm^{-1} and a negative band at ca. 250 cm^{-1} in Stokes. F: Time profiles of FSRS Stokes and anti-Stokes intensities in the center region of the ν(Ir-Ir) peak.

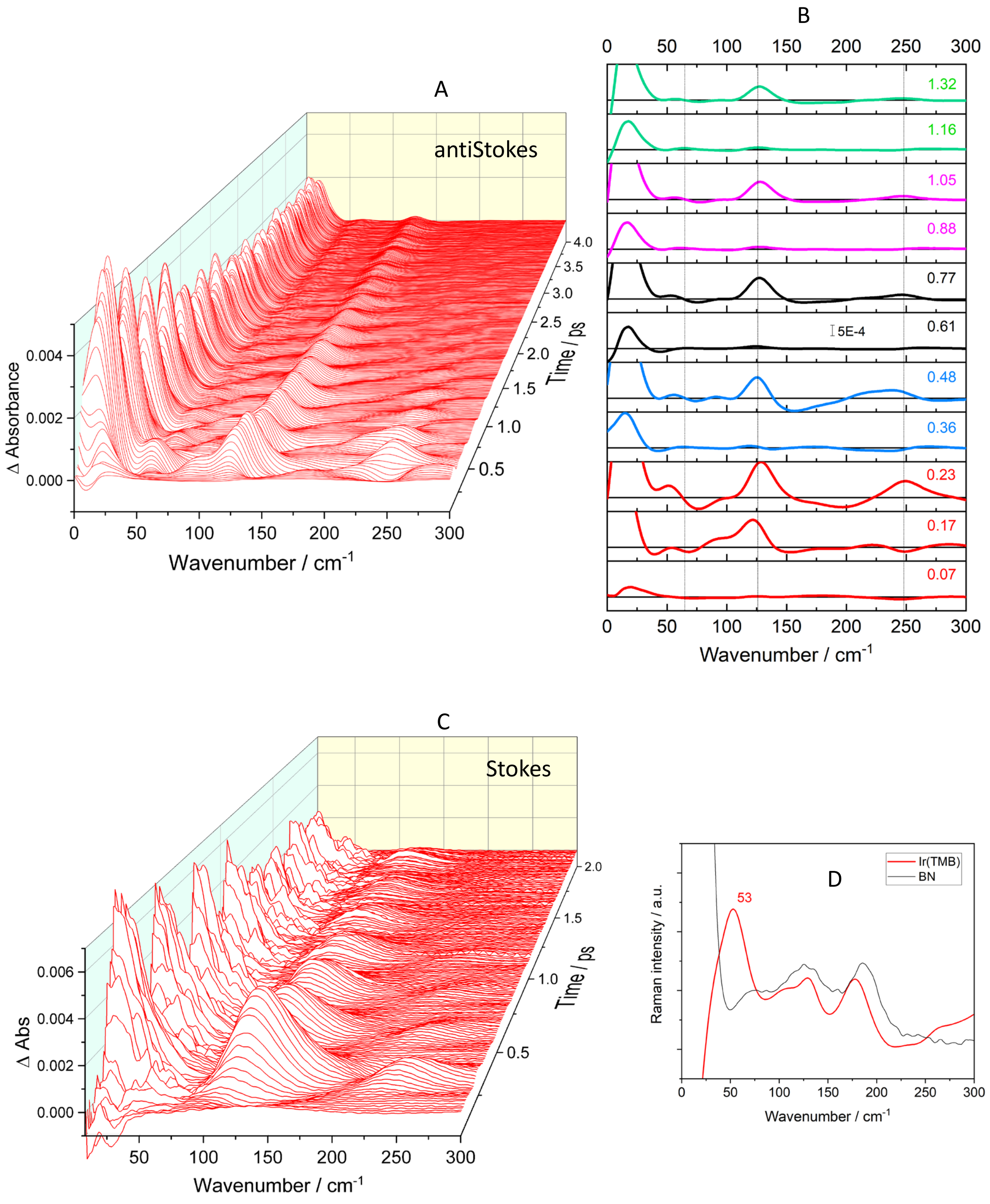

Figure S12. A-C: Time-resolved excited-state FSRS in AN. D: ground-state FSRS of Ir(TMB) in BN. A: spectral evolution of anti-Stokes spectra from 0.05 to 4.00 ps. B: selected spectra measured during the first five oscillation periods (distinguished by different colors). ΔAbs (y-axis) scale: -6.5×10^{-4} - 1.7×10^{-3}. Vertical dotted lines indicate the peak wavenumbers in the late-time spectra: 65, 126 cm^{-1}, and the ν(Ir-Ir) overtone at 248 cm^{-1}. C: spectral evolution of Stokes spectra from 0.04 to 2.00 ps.

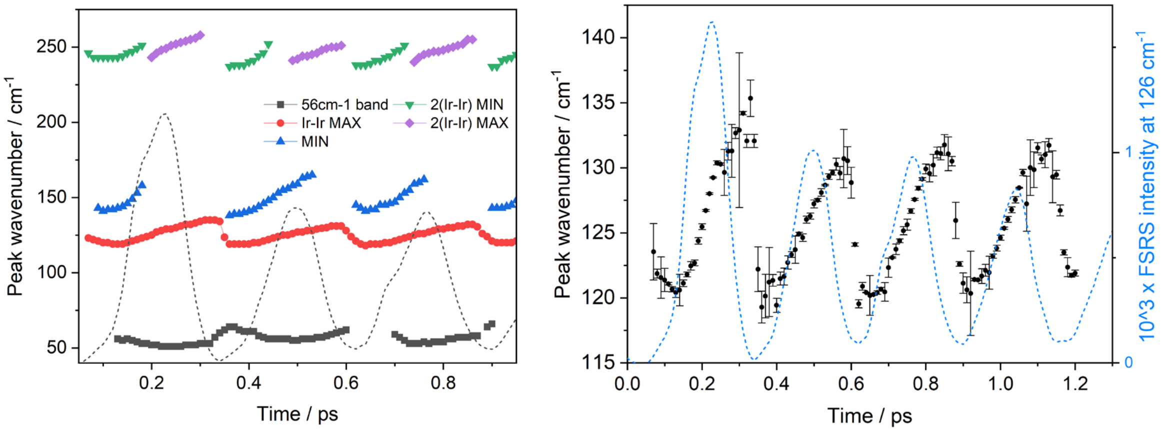

Figure S13. Time-dependent peak wavenumbers of selected anti-Stokes FSRS features in AN during the first three oscillation periods and at the beginning of the fourth, shown together with ν(Ir-Ir) 126 cm^{-1} intensity time profiles (dashed curves). Left: Values read directly from spectra. The positive band at ca. 55 cm^{-1} (black squares) kept shifting higher (while oscillating in a relatively narrow range) toward the final position of 65 cm^{-1} in late spectra. It was not observable between 0.61 and 0.69 ps, owing to overlap with a deepening negative minimum at ~40 cm^{-1}. The ν(Ir–Ir) peak (red circles) gradually increased from 119 to 135 cm^{-1} and then returned quickly to 119 cm^{-1} in the period of low peak intensity. The range of wavenumber oscillations decreased on going to later times. (peak wavenumbers shown could be affected by overlap with adjoining negative features at 140-160 cm^{-1} (blue).) The negative minima (green) and positive maxima (violet) of the ν(Ir–Ir) overtone feature shift higher within each period, approximately following the shifting fundamental. Right: ν(Ir-Ir) peak wavenumbers determined by gaussian shape fitting and their uncertainties.

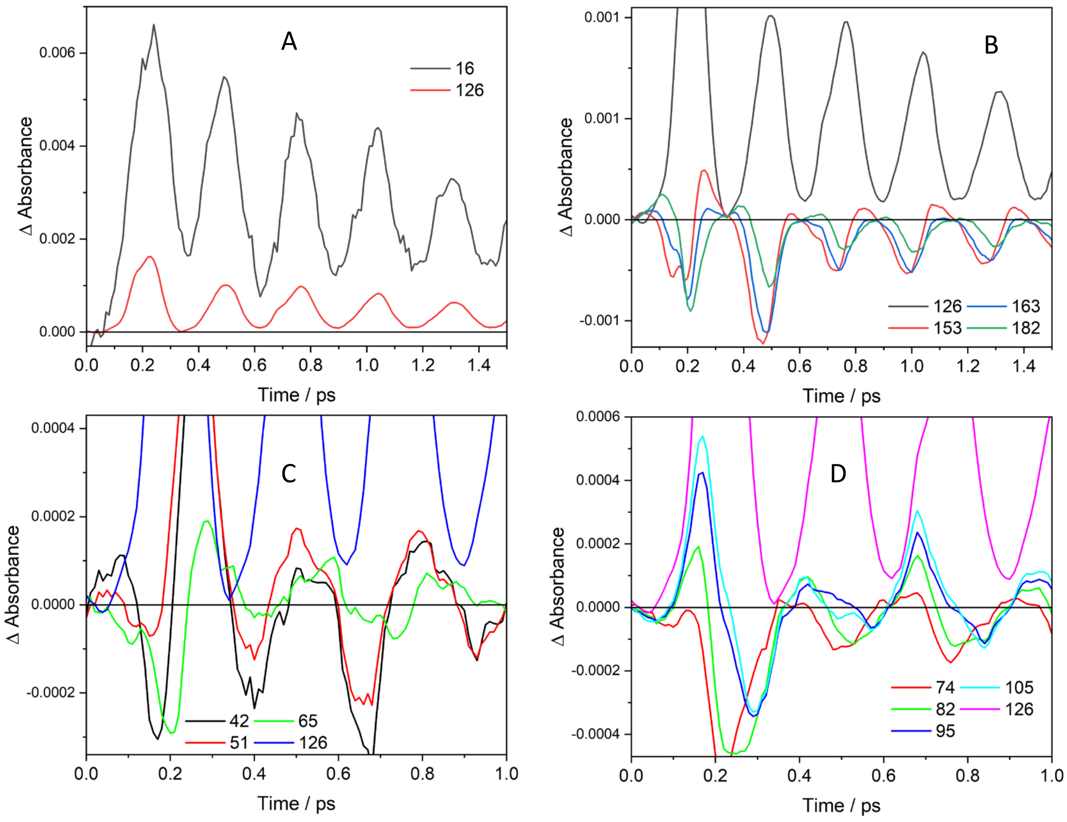

Figure S14. Time profiles at characteristic FSRS wavenumbers in AN. A: comparison of the 16 and 126 cm^{-1} features. B: profiles at the 126 cm^{-1} ν(Ir–Ir) peak and at selected higher wavenumbers. C: profiles of low-frequency features together with the ν(Ir–Ir) band. Profiles at 42 and 51 cm^{-1} show similar oscillation patterns but differ from that at 65 cm^{-1}. D: selected profiles at and below the ν(Ir–Ir) band. Profiles at 95-105 cm^{-1} show similar oscillation patterns but differ from that at 74-82 cm^{-1}. Comparison with panel C shows that oscillations at 65 and 74 cm^{-1} differ from each other.

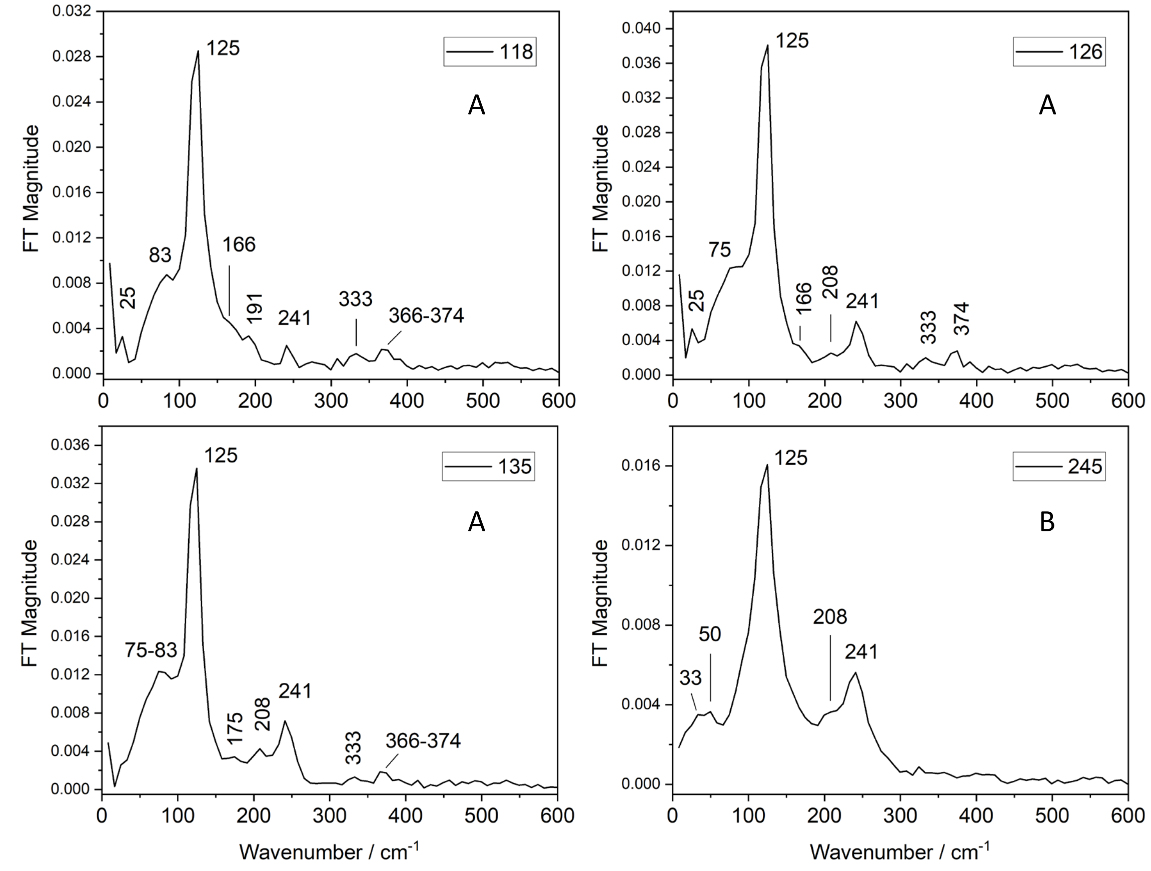

Figure S15. Fourier spectra of FSRS intensity oscillations across the ν(Ir-Ir) fundamental band (A) and at the overtone feature (B) measured at wavenumbers specified in the boxes top-right. Steps between FT points are 8.3 cm^{-1}.

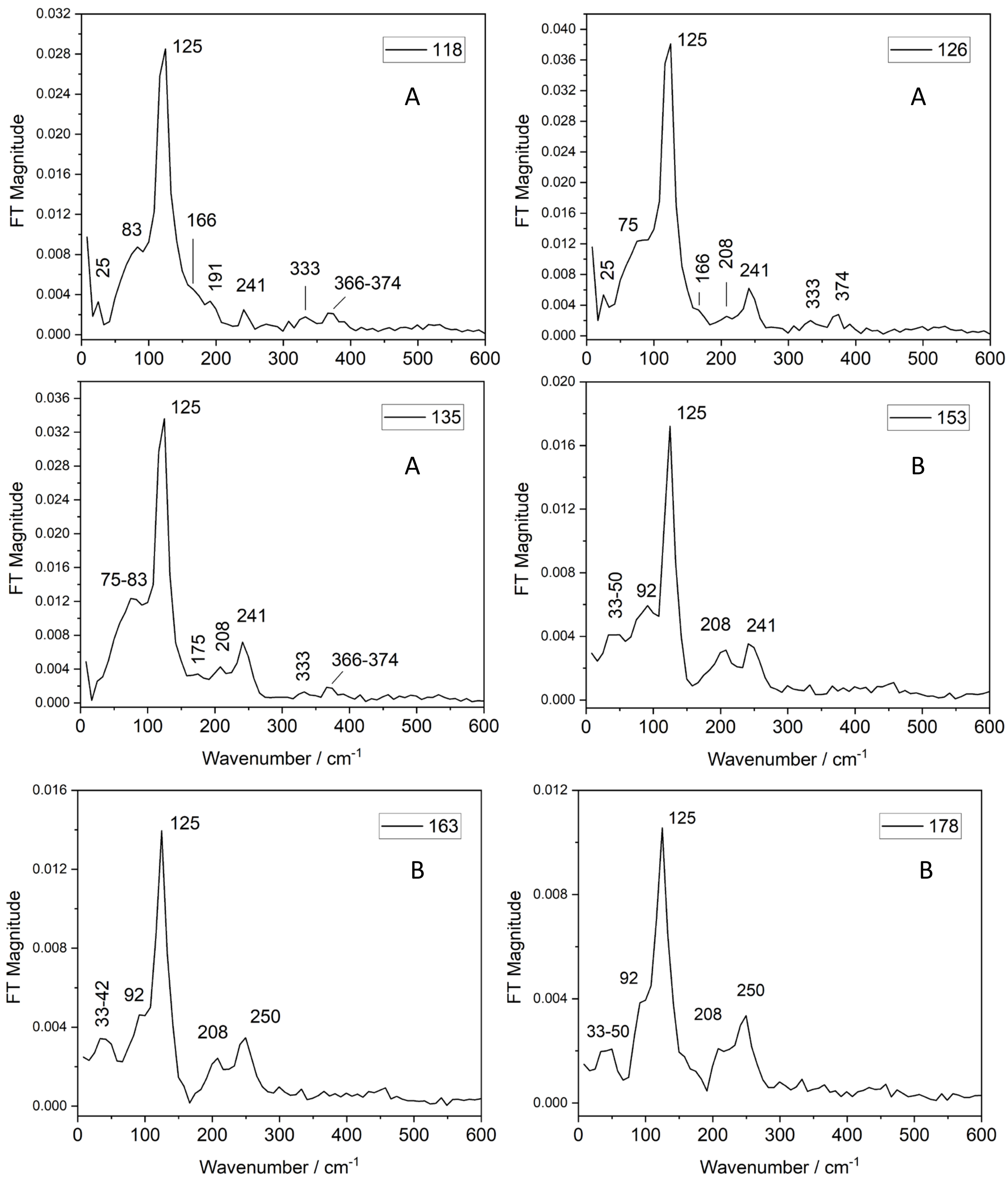

Figure S16. Fourier spectra of FSRS intensity oscillations across the ν(Ir-Ir) fundamental band (A) and the negative features at its blue side (B). Measured at wavenumbers specified in the boxes top-right. Steps between FT points are 8.3 cm^{-1}.

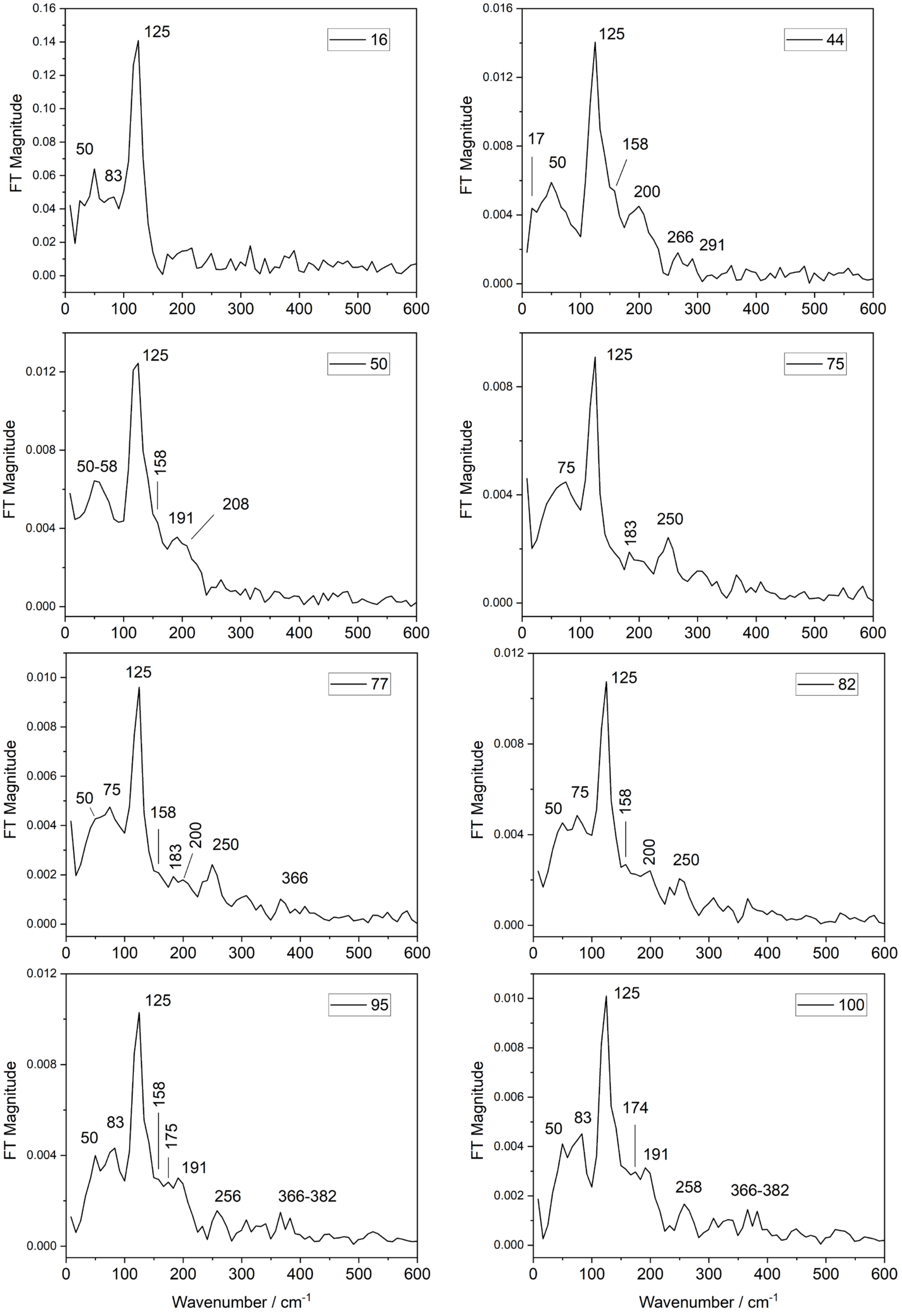

Figure S17. Fourier spectra of FSRS intensity oscillations in the low-wavenumber range.

Measured at wavenumbers specified in the boxes top-right. Step between points 8.3 cm^{-1}.

Note distinct patterns for 16 cm^{-1} (predominantly 120 cm^{-1}, contributions at higher wavenumbers of very low amplitudes), 44-60 cm^{-1} (medium-amplitude contributions of frequencies above and below the principal ~120 cm^{-1} peak), 75-77 cm^{-1} and 82-100 cm^{-1} (relatively large amplitudes at 50 and 70 cm^{-1}). Fourier spectra in the 82-100 cm^{-1} range also are distinct from those in the range of the ν(Ir–Ir) fundamental (118-135 cm^{-1}) shown in Figures S15 and S16.

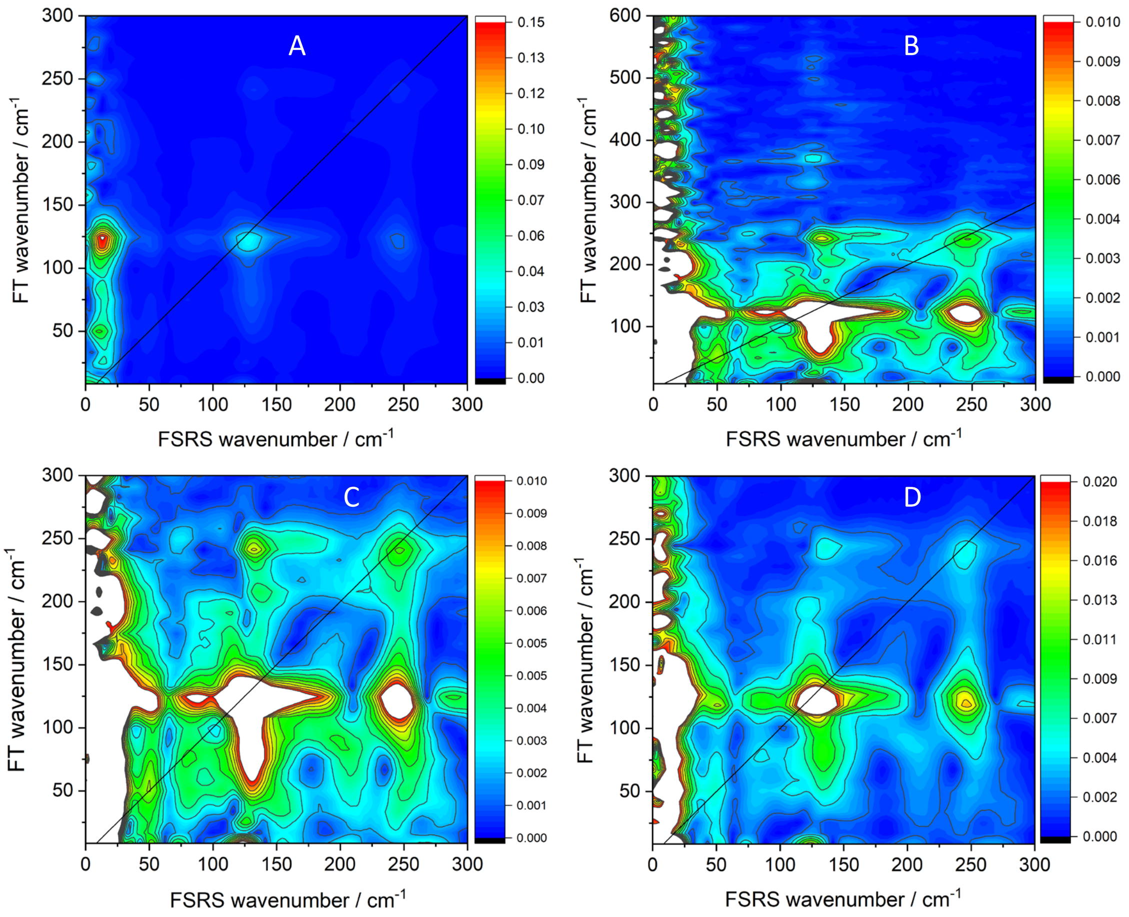

Figure S18. 2D FSRS of Ir(TMB) in AN. The *y*-axis shows wavenumbers of Fourier-transformed time-traces of FSRS intensities measured in 1 cm^{-1} steps along the *x*-axis. FT magnitudes are color-coded. Resolution at the *y* axis is 8.32 cm^{-1}. The diagonal is marked by the black line. Estimated peak positions are specified in the text and below in a FSRS×FT format. A: 2D spectrum obtained by FT transformation starting at 0 fs. FT-magnitude scale reduced to show the ν(Ir-Ir) diagonal peak at 128×125 cm^{-1}, its very strong cross-peak with the solute-solvent mode at 13×124 cm^{-1} and with the overtone at 245×124 cm^{-1}. Other cross-peaks involving the solute-solvent mode occur at 13×25, 11×51, and 12×83 cm^{-1}. B: expanded FT wavenumber and magnitude scales to show the cross-peak between the ν(Ir-Ir) fundamental and the 2nd overtone at 126×372 cm^{-1} and weaker cross-peaks at 124×333 cm^{-1}, 127×499 cm^{-1} (3rd overtone), 124×517 cm^{-1} (matches a weak ISRS feature), and 126×530 cm^{-1}. C: FT started at 0 fs, expanded magnitude scale (like in B) D: FT started at 60 fs, magnitude scale as in Figure 8 in the main text.

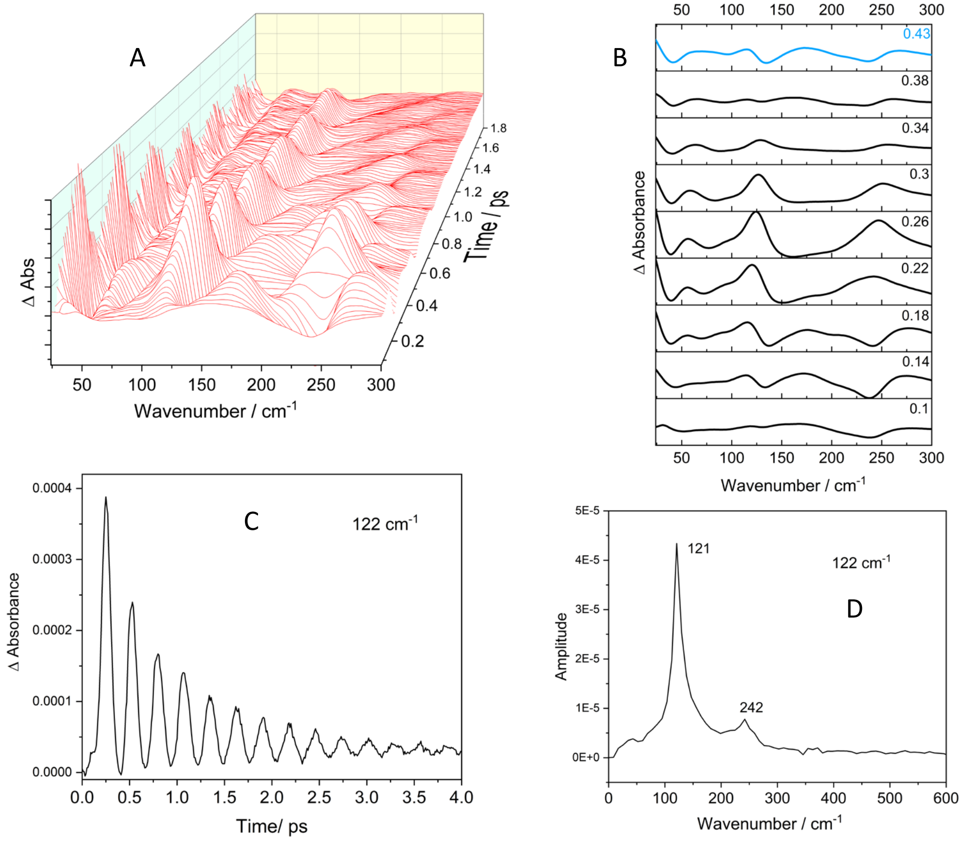

Figure S19. Overview of Ir(TMB) FSRS in BN. A: Waterfall representation of temporal evolution over the first 1.8 ps. Δ Abs from -1.7×10^{-4} to 4.0×10^{-4}. B: selected spectra from the 2nd oscillation period. Δ Abs from -2.2×10^{-4} to 3.9×10^{-4} in each panel. C: Signal intensity oscillations at 122 cm^{-1}. D: Fourier frequency spectrum of C.

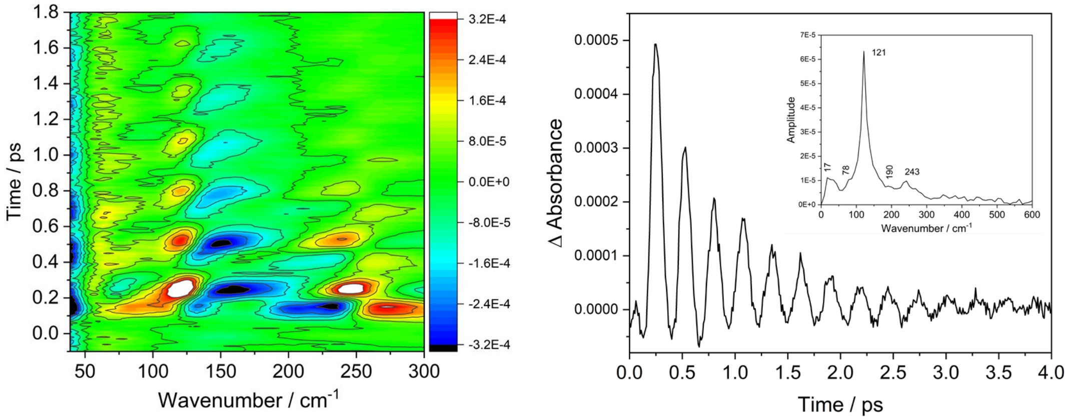

Figure S20. Overview of Ir(TMB) FSRS in THF. Left: Time × wavenumber map of spectra recorded over the first 1.8 ps. Right: Time profile of signal intensity at 122 cm^{-1}. Inset: Fourier frequency spectrum.

Experimental section

Sample synthesis and handling

2,5-diisocyano-2,5-dimethylhexane (TMB): TMB was prepared from 10 g of 2,5-diamino-2,5-dimethylhexane[4] *via* the carbylamine reaction, using a biphasic mixture of chloroform and sodium hydroxide, with benzyltriethylammonium chloride as a phase-transfer catalyst.[5] After quenching the reaction with ice water, the organic layers were separated and washed with 100-mL portions of 0.1 M sodium phosphate buffer, pH 5.5, until no amine starting material was observed by thin-layer chromatography. The resulting solution was dried over calcium sulfate, filtered, and rotovapped to dryness. Crude TMB was filtered through a silica gel column eluted with dichloromethane, dried, and recrystallized from ethanol. Isolated yield: 4.6 g (42%).

$[Ir(TMB)](BAr^F{}_4)_2$ ($BAr^F{}_4$ = $[\{3,5\text{-}(CF_3)_2C_6H_3\}_4B]^-$) was prepared and characterized as described in refs.[1, 6, 7] using $NaBAr^F{}_4$ instead of $NaBPh_4$. Solutions for spectroscopic measurements were made in degassed (SureSeal®) solvents (SigmaAldrich) and transferred into a sealed 1 mm Hellma cell under argon in a glove box. The sample position was scanned in the transversal plane during Raman/TA experiments. To ensure sample integrity, UV-vis absorption spectra were measured before and after each experiment.

Femtosecond Stimulated Raman Spectroscopy (FSRS)

All experiments were conducted on a homebuilt set-up constructed around femtosecond Titanium Sapphire amplifier Femtopower (Spectra Physics), generating 4.2 mJ pulses of ~20 fs duration at a repetition rate of 1 kHz and a Solstice amplifier (Spectra Physics), both sharing the fs oscillator (Element, SpectraPhysics). Amplifiers were synchronized both by means of electronic triggering and optical delay of the seed prior amplification. FSRS and TA experiment were integrated in one set-up as described below. The experiment was based on controlled time overlap of three pulses in the sample, denoted as Probe (PP -probing transient absorption and Raman transition), Raman pump (RP -driving sample into vibration coherence with the Probe), and Actinic pump (AP - triggering the desired photoreaction).

To generate probe pulses, ~0.4 mJ of the laser output was split and used to pump a two-stage optical parametric amplifier to obtain 1450 nm pulses of ~40 fs in duration. This output was used as a pump for a single-filament supercontinuum generated in a 2-mm CaF_2 plate, resulting in white light with a spectrum covering the spectral region from 370 nm to the near infrared (up to 1700 nm). The driving wavelength of 1450 nm was picked to have the undesired spike in the probe intensity from the white light driving pump matching the first peak of water infrared absorption. A 1 cm cuvette with water was then used as a very efficient notch filter, which removed the 1450 nm spike, resulting in a flat white light covering nearly the whole sensitivity range of the CCD detector. The probe was imaged onto the sample by a spherical mirror to a 50 µm diameter spot, and from the sample into the detection apparatus.

In the detection apparatus, the probe was split into two beams. One part was sent to a pair of a homebuilt grating-based high-resolution imaging spectrograph for Raman analysis in the Stokes region 800 to 1000 nm and anti-Stokes region 690 to 800 nm. The other part was directed to a prism spectrograph to obtain transient absorption spectra in 370 to 1200 nm range. In all three spectrographs, a 58x1024 pixels CCD camera (Entwicklungsbuero Stresing) was used as a linear image sensor via operation in a full vertical binning mode. Cameras were laser triggered at 1 kHz and provided full shot to shot detection with a dynamic range exceeding 30 000:1. Despite the low intensity of the single filament supercontinuum (~pJ/nm), it was possible to fully saturate the dynamic range of the sensors. At saturation level the readout noise of this detector was two orders of magnitude lower than the optical shot noise at given intensity, so all measurements can be considered only optical shot-noise limited.

The Raman pump was generated from ~1.5 mJ of the laser output transmitted via a 4f pulse shaper where a special disc with 96 shifted apertures was spinning at 10 Hz synchronized with the laser. Each aperture transmitted only one ~15 cm^{-1} interval of wavelengths at the time. This arrangement allowed production of 96 Raman pulses for each 100 incoming pulses, where each was ~5 cm^{-1} shifted from each other. Shifted signals were then numerically recombined. Such approach helped to reduce the fix-pattern noise and facilitate baseline correction. The Raman pulses were generated in the interval from 770 to 795 nm and resulting Raman spectra represented an average signal from all Raman measurements conducted over this interval. Four

pulses out of 100 were fully blocked to produce a pure transient absorption sequence along with the Raman experiment, leading to a cyclic scheme where the FSRS signal was measured for 96% of the time and pure TA signal was measured 4% of time. Raman pulses were guided via an optical delay line and then focused by a lens to a ~100 µm diameter spot overlapped with the probe. Temporal overlap between Raman and Probe pulses was adjusted to achieve maximal stimulated Raman gain while maintaining good spectral resolution. Average Raman pulse energy at the sample was ~3 µJ.

The actinic pulse was generated from 1.5 mJ of laser output pumping two stage OPA combined with sum frequency generation (TOPAS, Light Conversion). The 620 nm output was guided via a motorized optical delay line and focused into the sample via a lens. The actinic pulse diameter at the sample was ~100 µm at 620 nm. The actinic pulse energy was adjusted to 200 nJ. An actinic pulse train was guided via an optical chopper reducing its repetition rate to 500 Hz. The resulting experimental sequence was 96 FSRS experiments and 4 pure transmission measurements recorded for each 100 pulses, in both cases half of them pumped and half of them un-pumped. As a result, for each 100 laser pulses, 48 transient Raman experiments and 2 transient absorption experiments were conducted. Such approach is superior to the traditional method of using two ordinary choppers where FSRS and TA are measured at an equal fraction of time. It is more effective, since TA signals are typically orders of magnitude stronger and require much shorter acquisition times. The transient absorption signal was subtracted from the resulting Raman spectra and all spectrally shifted Raman signals were recombined based on the spectral calibration. Resulting spectra were individually baseline-treated by polynomial fitting of the baseline (including the solvent background) by the least absolute value residual method. We observed that such approach is less prone to artifacts than using the more common least square method. TA signals were chirp-corrected, whereas spectral chirp was neglected in the narrow Raman region of interest, 0-300 cm^{-1}. Most of the reported spectra were smoothed using the Savitzky-Golay method.

Impulsive Stimulated Raman Spectroscopy (ISRS)

ISRS were measured on a home-built setup constructed around a 1-kHz amplified Ti:sapphire laser system (Femtopower, Spectra Physics) that served as the primary source of all the pre-

pump, pump and probe beams. The 4-fs pump (400 nJ per pulse, focal $1/e^2$ –diameter: 160 µm) and probe (focal $1/e^2$ -diameter: 70 µm) beams originated from the white-light supercontinuum generated in argon-filled hollow-core fiber (Ultrafast Innovations) compressed by a chirp-mirror compressor. They spanned the spectral region between 500 and 1050 nm. The temporal pulse profiles were characterized by D-scan (Sphere Ultrafast Photonics). The timing between pump and probe pulses was scanned by the mechanical delay line with 6-fs steps up to 8 ps. The pre-pump beam (600 nm, 150 nJ per pulse, focal $1/e^2$ –diameter: 160 µm) was generated in NOPA (TOPAS, Light Conversion) and preceded the pump pulse by 300 fs. The probe beam transmitted through the sample was spectrally dispersed in a home-built dual-channel prism spectrometer and each pulse was detected by a 1-kHz CCD camera (Entwicklungsbuero Stresing). Probe spectral fluctuations negatively affecting the signal quality were corrected using the approach described in ref.[8]. All three beams were chopped by optomechanical choppers allowing for shot-to-shot basis detection of eight pulse configurations, from which the pump-probe (transient absorption) and prepump-pump-probe signals were retrieved. The probe and prepump beams were polarized identically, the polarization of the pump was turned by the magic angle.

Quantum Chemical Calculations

Electronic structures of Ir(TMB) in the ground- and lowest excited state were calculated by DFT using Gaussian 16 (G16) program package.[9] Geometry optimization of the lowest singlet excited state was performed by TDDFT. Raman frequencies and their scattering activities were calculated by vibrational analysis at optimized structures. Calculations were performed without any symmetry constraints for all investigated conformers and states. In order to obtain the approximate symmetries of vibrational modes, Ir(TMB) GS and excited-state structures were optimized within D_4 symmetry. DFT calculations employed the Perdew, Burke, Ernzerhof (PBE0) hybrid functional, [10, 11] with the GD3BJ version[12] of Grimme's dispersion added. AN solvent was described by the polarizable continuum model (PCM).[13] The following basis sets were used: double-ζ 6-31g basis set for H; polarized triple-ζ basis sets 6-311g(d)[14] for C, N and O atoms; and quasi-relativistic effective core pseudopotentials and a corresponding optimized set of Ir basis

functions.[15, 16] Reported vibrational frequencies were scaled by a factor of 0.956 that provided the best match with experimental ground-state ν(CN) bands.[2]

Wavepacket temporal evolution on the ^{1}dσ*pσ (S1) potential energy surface

Surfaces: The potential energy surfaces corresponding to Ir-Ir stretching were obtained by fitting energies calculated at the DFT and TDDFT level for the ground state (GS) and the excited state (S1), respectively, with the Morse potential[17]

$$V(x) = D(1 - e^{-a(r-r_0)})^2 + c \qquad \text{(eq. S1)}$$

where *D* is the depth of the potential well, r_0 is the distance corresponding to the potential minimum, and *a* is associated with the width of the potential well. Parameter c is used to see a mutual energy shift between the surfaces. The energies were evaluated for 30 (27) optimized geometries with a fixed Ir-Ir distance in the range 2.5 – 4.0 Å for GS (S1). The fitted parameters obtained using the Gnuplot program are set out in Table S4.

Table S4. Fitted parameters of the Morse potential for the GS and S1 state.

	**GS**	**S1**
***D* (eV)**	0.2776 ± 0.0026	1.5253 ± 0.0088
***a* (Å^{-1})**	1.7059 ± 0.0069	1.3397 ± 0.0067
***r_0* (Å)**	3.16126 ± 0.00091	2.87791 ± 0.00055
***c* (eV)**	0.0000 ± 0.0005	1.6917 ± 0.0008

To find solution for vibrational energies

$$E_n = h\nu_0(n + 1/2) - \frac{[h\nu_0(n + 1/2)]^2}{4D}, \nu_0 = \frac{a}{2\pi}\sqrt{\frac{2D}{m_{eff}}} \qquad \text{(eq. S2)}$$

and vibrational wavefunctions

$$\psi_n(z) = N_n z^{\lambda - n - \frac{1}{2}} e^{\frac{-1}{2}z} L_n^{(2\lambda - 2n - 1)}(z)\,, \qquad \text{(eq. S3)}$$

where

$$z = 2\lambda e^{-a(r-r_0)}, \lambda = \frac{\sqrt{2m_{eff}D}}{a\hbar}, N_n = [\frac{n!(2\lambda-2n-1)a}{\Gamma(2\lambda-n)}]^{\frac{1}{2}}, \quad \text{(eq. S4)}$$

the effective mass m_{eff} of the vibration was half the Ir atomic mass, i.e. 175,198 a.u. (reduced Ir_2 mass). The estimation was in line with an experimentally determined frequency of 122 cm^{-1} for S1 and fitted well depth from equation S2, which gives 178,687 a.u. This estimate led to 66 bound states for GS and 198 for S1 since their number is limited by the greatest integer less than or equal to $\lambda - 1/2$. Energies of fifteen lowest levels are given in Table S5.

Table S5. GS and S1 vibrational energies (in cm^{-1}).

	GS		S1	
**n**	**E_n**	**$E_n - E_{n-1}$**	**E_n**	**$E_n - E_{n-1}$**
0	33.8		62.3	
1	100.5	66.8	186.4	124.1
2	166.2	65.7	309.9	123.5
3	231.0	64.7	432.8	122.9
4	294.6	63.7	555.0	122.2
5	357.3	62.7	676.6	121.6
6	418.9	61.6	797.6	121.0
7	479.6	60.6	917.9	120.3
8	539.2	59.6	1037.7	119.7
9	597.7	58.6	1156.7	119.1
10	655.3	57.5	1275.2	118.4
11	711.8	56.5	1393.0	117.8
12	767.3	55.5	1510.2	117.2
13	821.8	54.5	1626.7	116.6
14	875.2	53.5	1742.7	115.9

Wavepacket and its development: The initial population on S1 was generated based on Franck-Condon factors (FC), i.e., squares of overlap integrals between the GS wavefunction (wavepacket) and S1 vibrational levels that gave probabilities of individual vibrational levels contained in the wavepacket. Owing to the form of wavefunctions for the Morse potential, numerical integration was employed in the Mathematica 14 program. Because of the presence of gamma functions and exponentials with large arguments in analytic form of eq. S4, the calculation was reduced to evaluation of overlap integrals S(0,n') between the GS 0th vibrational level and 14 lowest

vibrational levels of the excited state (Table S6) due to numerical reasons. In this way, GS thermal population (according to Boltzmann statistics) was not considered. Turning to the initial wavepacket description on the S1 state (t = 0),

$$\Phi(z, t = 0) = \sum_{n'} S(0, n')\psi_{n'}(z) \tag{eq. S5}$$

the wavefunction at time τ was obtained using the standard formalism of quantum mechanics,

$$\Phi(z, t = \tau) = \sum_{n'} S(0, n')e^{\frac{-iE_{n'}\tau}{\hbar}}\psi_{n'}(z). \tag{eq. S6}$$

Note that the S1 population decrease was neglected.

Table S6. Calculated Franck-Condon (FC) factors and overlaps (S(0,n')) between the GS 0th vibrational level and the S1 n^{th} vibrational level.

n'	S(0,n')	FC(0,n')
0	0.000	0.000
1	0.000	0.000
2	0.076	0.006
3	0.116	0.013
4	0.158	0.025
5	0.200	0.040
6	0.237	0.056
7	0.267	0.071
8	0.289	0.083
9	0.300	0.090
10	0.302	0.091
11	0.293	0.086
12	0.271	0.073
13	0.157	0.025
14	-0.775	0.601

References

1. Smith, D. C. Electronic Structure and Photochemical Reactivity of Binuclear Metal Complexes. Ph.D. Dissertation, California Institute of Technology, Pasadena, California, 1989.
2. Pižl, M.; Hunter, B. M.; Sazanovich, I. V.; Towrie, M.; Gray, H. B.; Záliš, S.; Vlček, A., Excitation-Wavelength-Dependent Photophysics of d^8d^8 Di-isocyanide Complexes. *Inorg. Chem.* **2022,** *61*, 2745-2759.
3. Smith, D. C.; Miskowski, V. M.; Mason, W. R.; Gray, H. B., Electronic Absorption and MCD Spectra of $M_2(TMB)_4^{2+}$, M = Rh and Ir. A Valence-Bond Description of the Upper Electronic Excited States. *J. Am. Chem. Soc.* **1990,** *112*, 3759-3767.
4. Coffman, D. D.; Jenner, E. L.; Lipscomb, R. D., Syntheses by Free-radical Reactions. I. Oxidative Coupling Effected by Hydroxyl Radicals. *J. Am. Chem. Soc.* **1958,** *80*, 2864-2872.
5. Gokel, G. W.; Widera, R. P.; Weber, W. P., Phase-transfer Hofmann Carbylamine Reaction: tert-Butyl Isocyanide. *Org. Synth.* **1976,** *55*.
6. Smith, T. P. Syntheses and Characterization of a Series of Binuclear Iridium Complexes. Ph.D. Dissertation, California Institute of Technology, Pasadena, California, 1982.
7. Miskowski, V. M.; Smith, T. P.; Loehr, T. M.; Gray, H. B., Properties of Metal-Metal Single Bonds. Vibrational and Electronic Spectra of Binuclear Rhodium(II) and Iridium(II) Isocyanide Complexes with Comparisons to $Mn_2(CO)_{10}$. *J. Am. Chem. Soc.* **1985,** *107*, 7925-7934.
8. Feng, Y.; Vinogradov, I.; Ge, N.-H., General noise suppression scheme with reference detection in heterodyne nonlinear spectroscopy. *Opt. Express* **2017,** *25*, 26262-26279.
9. Frisch, M. J.; Trucks, G. W.; Schlegel, H. B.; Scuseria, G. E.; Robb, M. A.; Cheeseman, J. R.; Scalmani, G.; Barone, V.; Petersson, G. A.; Nakatsuji, H.; Li, X.; Caricato, M.; Marenich, A. V.; Bloino, J.; Janesko, B. G.; Gomperts, R.; Mennucci, B.; Hratchian, H. P.; Ortiz, J. V.; Izmaylov, A. F.; Sonnenberg, J. L.; Williams-Young, D.; Ding, F.; Lipparini, F.; Egidi, F.; Goings, J.; Peng, B.; Petrone, A.; Henderson, T.; Ranasinghe, D.; Zakrzewski, V. G.; Gao, J.; Rega, N.; Zheng, G.; Liang, W.; Hada, M.; Ehara, M.; Toyota, K.; Fukuda, R.; Hasegawa, J.; Ishida, M.; Nakajima, T.; Honda, Y.; Kitao, O.; Nakai, H.; Vreven, T.; Throssell, K.; J. A. Montgomery, J.; Peralta, J. E.; Ogliaro, F.; Bearpark, M. J.; Heyd, J. J.; Brothers, E. N.; Kudin, K. N.; Staroverov, V. N.; Keith, T. A.; Kobayashi, R.; Normand, J.; Raghavachari, K.; Rendell, A. P.; Burant, J. C.; Iyengar, S. S.; Tomasi, J.; Cossi, M.; Millam, J. M.; Klene, M.; Adamo, C.; Cammi, R.; Ochterski, J. W.; Martin, R. L.; Morokuma, K.; Farkas, O.; Foresman, J. B.; Fox, D. J. *Gaussian16, Revision C.01*, Gaussian, Inc.: Wallingford, CT, 2019.
10. Perdew, J. P.; Burke, K.; Ernzerhof, M., Generalized Gradient Approximation Made Simple. *Phys. Rev. Lett.* **1996,** *77*, 3865-3868.
11. Adamo, C.; Barone, V., Toward reliable density functional methods without adjustable parameters: The PBE0 model. *J. Chem. Phys.* **1999,** *110*, 6158-6170.
12. Grimme, S.; Ehrlich, S.; Goerigk, L., Effect of the damping function in dispersion corrected density functional theory. *J. Comput. Chem.* **2011,** *32*, 1456-1465.
13. Tomasi, J.; Mennucci, B.; Cammi, R., Quantum Mechanical Continuum Solvation Models. *Chem. Rev.* **2005,** *105*, 2999-3093.
14. Raghavachari, K.; Binkley, J. S.; Seeger, R.; Pople, J. A., Self-consistent molecular orbital methods. XX. A basis set for correlated wave functions *J. Chem. Phys.* **1980,** *72*, 650-654.

15. Andrae, D.; Häussermann, U.; Dolg, M.; Stoll, H.; Preuss, H., Energy-Adjusted ab initio Pseudopotentials for the Second and Third Row Transition Elements. *Theor. Chim. Acta* **1990,** *77*, 123-141.
16. Martin, J. M. L.; Sundermann, A., Correlation Consistent Valence Basis Sets for Use with the Stuttgart–Dresden–Bonn Relativistic Effective Core Potentials: The Atoms Ga–Kr and In–Xe. *J. Chem. Phys.* **2001,** *114*, 3408-3420.
17. Dahl, J. P.; Springborg, M., The Morse oscillator in position space, momentum space, and phase space. *J. Chem. Phys.* **1988,** *88*, 4535–4547.
